# Sensory and motor contents are prioritized dynamically in working memory

**DOI:** 10.1371/journal.pbio.3003273

**Published:** 2025-07-14

**Authors:** Irene Echeverria-Altuna, Sage E. P. Boettcher, Freek van Ede, Anna C. Nobre

**Affiliations:** 1 Department of Experimental Psychology, University of Oxford, Oxford, United Kingdom; 2 Department of Psychology, Yale University, New Haven, Connecticut, United States of America; 3 Institute for Brain and Behavior Amsterdam, Department of Experimental and Applied Psychology, Vrije Universiteit Amsterdam, Amsterdam, The Netherlands; Peking University, CHINA

## Abstract

Internal selective attention prioritizes both sensory and motor contents in working memory to guide prospective behavior. Prior research has shown how attention modulation of sensory contents is flexible and temporally tuned depending on access requirements, but whether the prioritization of motor contents follows similar flexible dynamics remains elusive. Also uncharted is the degree of co-dependence of sensory and motor modulation, which gets at the nature of both working-memory representations and internal attention functions. To address these questions, we independently tracked the prioritization of sensory and motor working-memory contents as a function of dynamically evolving temporal expectations in human participants. The design orthogonally manipulated when an item location (left versus right side) and associated prospective action (left versus right hand) would be relevant. Contralateral-vs-ipsilateral modulation of posterior alpha (8–12 Hz) activity in electroencephalography (EEG) tracked prioritization of the item location, while contralateral-vs-ipsilateral modulation of central mu/beta (8–30 Hz) activity tracked response prioritization. Proactive and dynamic alpha and mu/beta modulation confirmed the flexible and temporally structured prioritization of sensory and motor contents alike. Intriguingly, the prioritization of sensory and motor contents was temporally uncoupled, showing dissociable patterns of modulation. The findings reveal multiple modulatory functions of internal attention operating in tandem to prepare relevant aspects of internal representations for adaptive behavior.

## Abstract 2

Attention flexibly modulates sensory contents in working memory, but the dynamics of motor content modulation in working memory remain unclear. This study shows that brain representations of both sensory and motor contents in working memory are modulated by attentional shifts, and are temporally uncoupled from one another.

## Introduction

Internal selective attention selects and prioritizes working-memory contents according to goals, expectations, and other control-related cognitive processes [1–6]. Far from changing randomly, our environment is structured across space, time, and other attributes (e.g., [7,8]). Internal attention can use the expectations emerging from these regularities to prioritize working-memory contents and, thereby, proactively guide ongoing behavior.

Numerous studies have found that expectations about the sensory qualities of objects (i.e., location, color, and other stimulus features) can proactively guide internal attention to the relevant working-memory contents thus facilitating subsequent task performance ([5,9,10–12]; for reviews see [6,13]). A growing literature has also highlighted the pragmatic nature of internal attention, demonstrating that it can proactively prioritize action-related contents in working memory [14–23]. Sensory and action-related contents can co-exist in working memory. When a working-memory item is probed, selection of its sensory- and motor-associated contents starts concurrently [23]. Beyond this initial demonstration, the dynamics of the prioritization of sensory and motor contents that co-exist in working memory remain elusive.

Electroencephalography (EEG) provides a powerful method for independently tracking the prioritization of sensory- and action-related contents in working memory. The selection of action plans in working memory is mirrored by a relative reduction in mu/beta (8–30 Hz) activity contralateral to the prospective hand action in central electrodes (e.g., [22–24,25,26,27]). In turn, the selection of visual representations held within the spatial layout of working memory is mirrored by a relative reduction in contralateral alpha-frequency (8–12 Hz) activity in posterior electrodes [23,28–33]. Lateralized modulations in posterior alpha and sensorimotor mu/beta activity related to location and action prioritization, respectively, have been proposed to reflect changes in the excitability of the underlying neural sources [24,25,26,34–38].

It is increasingly clear that the prioritization of sensory contents in working memory is highly flexible and dynamic. For example, selecting a sensory item in working memory does not necessarily compromise the representation of other competing items, illustrating the flexible and reversible nature of working-memory content prioritization [9,39–48]. Moreover, the prioritization of sensory contents, as measured with EEG activity modulations or pupil size, changes according to temporal expectations concerning the likely time of an item to be probed [32,41,49–51]. In contrast, it remains unknown whether the prioritization of action-related working-memory contents can be similarly reversible and tuned to the temporal structure of the task. Additionally, while sensory- and action-related working-memory contents can be prioritized concurrently [23], it is unclear whether these two aspects of the representation are functionally bound. Are they two sides of the same integrated internal representation and, therefore, does their prioritization co-evolve in lockstep according to the dynamic structure of the task? Or are sensory and motor contents in working memory dissociable and capable of separate modulation?

To address these questions, we designed a task that zoomed into how temporal expectations guide the dynamic prioritization of sensory- and action-related contents in working memory. Item location (left versus right side) was orthogonally crossed with required action (left versus right hand). The design thereby enabled tracking the prioritization of sensory contents (location) and prospective actions independently (see also [14,19,23]). Contralateral-vs-ipsilateral posterior alpha (8–12 Hz) and central mu/beta (8–30 Hz) modulations provided markers of the prioritization of sensory- and action-related contents in working memory, respectively. To foreshadow the results, the prioritization of both sensory- and action-related working-memory contents evolved flexibly as a function of dynamically changing expectations. However, their prioritization was not continuously temporally coupled, suggesting that multiple modulatory functions can operate in tandem – and develop independently – on different aspects of working-memory representations.

## Results

The present study investigated whether and how sensory and motor working-memory contents could be prioritized as a function of dynamically evolving expectations. With this aim, participants completed a visual-motor working-memory task in which retro-cues and dynamically changing expectations guided the prioritization of sensory- and action-related working-memory contents.

In each trial, participants viewed two colored, tilted bars. They had to report the tilt of one of the bars at the end of each trial. In half of the trials (informative), retro-cues that matched the color of one of the encoded bars indicated the item participants had to report at the end of the trial. In noninformative trials (50%), retro-cues with non-matching colors were uninformative about the to-be-probed item. Trials varied in duration, with the probe appearing either *early* (1 s) or *late* (3 s) following the retro-cue. In informative trials, the to-be-reported item was determined by both the color of the retro-cue and the length of the delay between the cue and the probe. On short-delay trials, participants were probed to report the tilt of the bar matching the retro-cue color. On long-delay trials, they were probed to report the tilt of the *other* bar. In noninformative trials, the to-be-reported item was unpredictable.

The location of the bars on the screen (left versus right) and their orientation (leftward versus rightward tilt) were manipulated independently. Importantly, bar orientation (left versus right) dictated the hand (left versus right) required for responding. The orthogonal manipulation of item location and tilt enabled the independent tracking of the EEG markers of sensory- and action-related content prioritization in working memory. Lateralization of posterior alpha-band activity tracked the prioritization of the spatial location of an item in working memory, a sensory attribute that was unrelated to the required response. Lateralization of central mu/beta-band activity tracked the prioritization of the response hand associated with an item in working memory, based on the orientation of the item, independent of its location.

### Behavioral results

**Fig 1:**
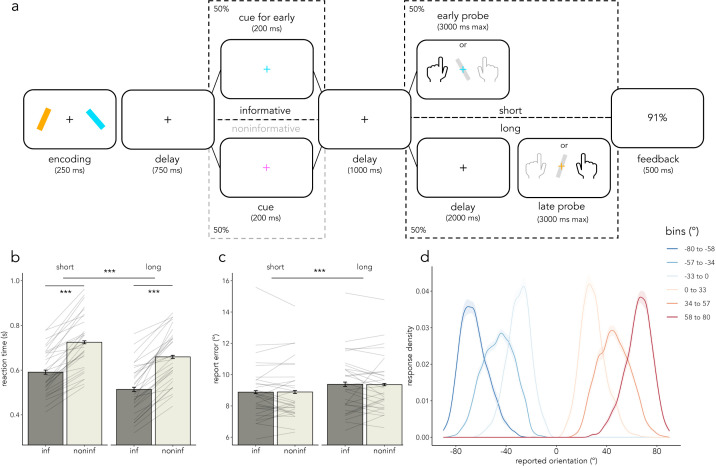
Task design and behavioral results. (a) Trial schematic. Two colored, tilted bars (one on the left and the other on the right, one tilted to the left and the other to the right, with location and tilt being orthogonally manipulated) were displayed at encoding. Participants were probed to report the orientation of one of the two encoded bars at the end of each trial. In half of the trials (informative) a retro-cue matching the color of one of the two bars was shown; in noninformative trials (50%), a cue with a different color appeared instead. In informative trials, if the delay after cue offset was short (1 s), participants were probed about the cued item. Alternatively, if the delay was long (3 s), they had to report the other (uncued) item. In noninformative trials the probed item was unpredictable. Participants received feedback about their response accuracy on that trial as a percentage. Reaction time (s; panel **b**) and report error (°; panel **c**) are plotted in informative and noninformative and short and long trials. Thin grey lines represent individual participants (*N* = 30), error bars represent the standard error of the mean (SEM), light grey represents noninformative trials, and dark grey depicts informative trials. (d) Average response density as a function of the reported orientation and in relation to the orientation of the reported bar. Shaded areas represent SEM (*N* = 30). The data in this figure can be found in OSF under *data/behav* [52]. The numerical RT and report error values displayed in **(b)** and **(c)** can be found in S1 and S2 Data, respectively.

If participants prioritized each of the relevant working-memory contents at the expected times, their responses were hypothesized to be faster in both short and long informative trials compared to noninformative trials. A repeated-measures 2 × 2 ANOVA of RT with informativeness and duration as factors revealed a main effect of informativeness on RT (*F*(*1*,*29*) = 183.37, ***p < .001, *η*^2^ = .28), a main effect of duration on RT (*F*(*1*,*29*) = 101.17, ***p < .001, *η*^2^ = .09), and no interaction between the factors (*F*(*1*,*29*) = .78, *p* = .38, *η*^2^ < .000; Fig 1B; see also S1 and S3 Tables and S1 Data). Overall, participants were faster at responding to targets in informative trials than in noninformative trials, suggesting that both retro-cues and internally driven temporal expectations facilitated performance in this task. Participants were also faster in long trials compared to short trials, as expected from related foreperiod effects [53].

Report errors in this task were consistently low and insensitive to the informativeness of the retro-cue (*F*(*1*,*29*) = .003, *p* = .95, *η*^2^ < .001). A main effect of duration on error indicated smaller errors (higher accuracy) on short trials than long trials (*F*(*1*,*29*) = 24.25, ***p < .001, *η*^2^ = .02). The factors did not interact (*F*(*1*,*29*) = .02, *p* = .9, *η*^2^ < .001; Fig 1C; see also S2 and S4 Tables and S2 Data).

Although informativeness did not have a significant effect on report error, we confirmed that participants used orientation-related visual information to guide their responses by investigating the profile of the orientation reports. Namely, we found that participants’ responses varied systematically with the orientation of the probed bar (Fig 1D), rather than simply reflecting categorical left/right button presses. This confirms that participants were using the details of the visual representation in working memory to guide their reports.

### Alpha- and mu/beta-frequency activity modulation

**Fig 2:**
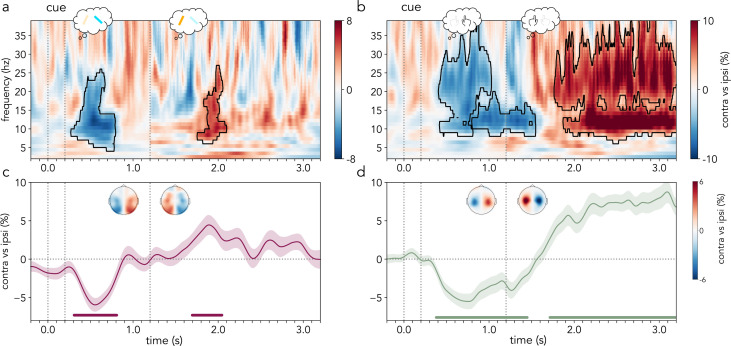
Lateralized frequency-specific EEG activity locked to cue onset. **(a)** Contrast between EEG time–frequency activity contralateral vs. ipsilateral to the cued bar location in occipital sensors (PO7/PO8) divided by summed contralateral and ipsilateral activity (expressed as a percentage) in informative trials vs. noninformative trials. **(b)** Contrast between EEG time–frequency activity contralateral vs. ipsilateral to the cued prospective action in central sensors (C3/C4) divided by summed contralateral and ipsilateral activity (expressed as a percentage) in informative trials vs. noninformative trials. **(c, d)** Cross-participant average alpha (8−12 Hz; **c**) and mu/beta (8−30 Hz; **d**) activity difference between contralateral and ipsilateral sensors to the cued location and action, respectively, in informative trials. Topographies represent the average frequency-specific activity in contra-vs-ipsi contrasts in informative trials across all sensor pairs during the time-windows which correspond to the alpha clusters in panel **c** or mu/beta clusters in panel **d**, respectively. Black outline in time–frequency spectra **(a, b)** indicates statistically significant clusters. Shaded areas represent the SEM and cluster-permutation corrected significant time points are indicated with horizontal lines in **c** and **d** (*N* = 30). The first part of the time–frequency spectra in panels **a** and **b** and of the time course in **c** and **d** (−0.2–1.2 s) corresponds to the average of short and long trials, and the second part (1.2–3.2 s) corresponds to long trials only. The vertical dotted lines represent (from left to right) the onset (0 s) and offset (0.2 s) of the retro-cue and the time of probe appearance in early trials (1.2 s). The data in this figure can be found in OSF under *data/eeg/trf* [52].

Next, we turned to the pre-defined alpha- and mu/beta-frequency EEG activity patterns in the delay after the retro-cue. Participants were hypothesized to initially prioritize the sensory- and action-related attributes of the cued item and, in cases where the duration of the short interval passed (long trials), to then prioritize the attributes of the other item. Based on previous studies (e.g., [33]), alpha activity (8–12 Hz) contralateral-vs-ipsilateral to the selected item location was hypothesized to be modulated by the internal prioritization of locations, despite the fact that location was not strictly necessary for the task [28,54–57]. Additionally, mu/beta activity (8–30 Hz) contralateral-vs-ipsilateral to the prospective hand action was predicted to change together with prospective action selection [23,26,24,31]. We utilized noninformative trials – in which participants could not reliably select specific sensory or response-related information before the probe – as a baseline for the selection signals in informative trials. Specifically, participant-wise cluster-based permutation analyses of the time–frequency spectra in the contralateral-versus-ipsilateral contrasts of visual and motor selection in informative versus noninformative trials confirmed our hypotheses (Fig 2A and 2B; see also S1 Fig).

A pronounced modulation in alpha activity contralateral-vs-ipsilateral to the selected item location was observed following the retro-cue in informative trials during the early part of the delay, which contained both short and long trials (Fig 2A; first cluster: ***p < .001, time range: 0.31–0.81 s, frequency range: 5–25 Hz, max lateralization time: 0.53 s, max lateralization frequency: 11 Hz). Strikingly, in long trials, the side of relative alpha attenuation shifted after the short interval had elapsed (Fig 2A; second cluster: *p = .03, time range: 1.76–2.1 s, frequency range: 7–26 Hz, max lateralization time: 1.94 s, max lateralization frequency: 7 Hz). This “shift” in relative alpha attenuation from one hemisphere to the other, pointed to a prioritization of each working-memory item location in turn. Initially, the cued item that was expected to be probed early was prioritized (Fig 2C; first cluster: ***p < .001, time range: 0.31–0.81 s). With the passage of time, relative priority shifted to the location of the other item, which was expected to be probed late (Fig 2C; second cluster: *p = .04, time range: 1.7–2.04 s).

In addition to the modulation of contralateral-vs-ipsilateral alpha activity over occipital EEG channels, the prioritization of stimulus locations in visual working memory has also been shown to be related to small biases in gaze position in the direction of internally selected items (e.g., [47,58,59]). The present study revealed a significant bias of gaze position towards the location of the cued item first, followed by a bias of gaze position towards the opposite location in long trials (S2 Fig). To confirm that the observed lateralized alpha modulation was not solely due to systematic changes in gaze position, alpha modulation was compared in trials with versus without gaze biases towards the prioritized locations. In line with previous studies showing a functional but non-obligatory link between gaze bias and alpha modulation [59], the additional analysis revealed comparable lateralized alpha modulations in trials with or without systematic gaze biases.

In parallel to sensory prioritization, a noticeable early reduction in mu/beta activity occurred in contralateral-vs-ipsilateral motor sensors according to the expected response hand in short trials (Fig 2B; first cluster: **p = .003, time range: 0.41–1.02 s, frequency range: 9–39 Hz, max lateralization time: 0.76 s, max lateralization frequency: 12 Hz; second cluster: *p = .04, time range: 0.8–1.54 s, frequency range: 9–19 Hz, max lateralization time: 1.24 s, max lateralization frequency: 12 Hz). Strikingly, the side of relative mu/beta attenuation also shifted from one hemisphere to the opposite, mirroring the flexible prioritization of action plans corresponding to the first and second items, respectively (Fig 2B; only long trials; third cluster: ***p < .001, time range: 1.77–3.2 s, frequency range: 7–39 Hz, max lateralization time: 2.6 s, max lateralization frequency: 36 Hz). These findings were confirmed on the average time course of lateralized mu/beta activity (Fig 2D; first cluster: **p = .005, time range: 0.38–1.45 s; second cluster: ***p < .001, time range: 1.72–3.2 s). Importantly, the present findings were not dependent upon EEG sensor choice as a larger set of visual and motor channels revealed an equivalent pattern of results (S3 Fig).

Similar to previous studies [23], the modulation of lateralized alpha activity linked to the prioritization of item location had a lateralized occipital (visual) EEG topography (Fig 2C), while changes in lateralized mu/beta activity were predominantly confined to central (motor) EEG channels (Fig 2D). The prioritization of the first and second location modulated alpha-frequency activity with comparable topographies. Similarly, the prioritization of the first and second prospective actions had similar topographies and modulated a similar mu/beta frequency band.

### Temporal coupling between lateralized alpha and mu/beta modulation

**Fig 3:**
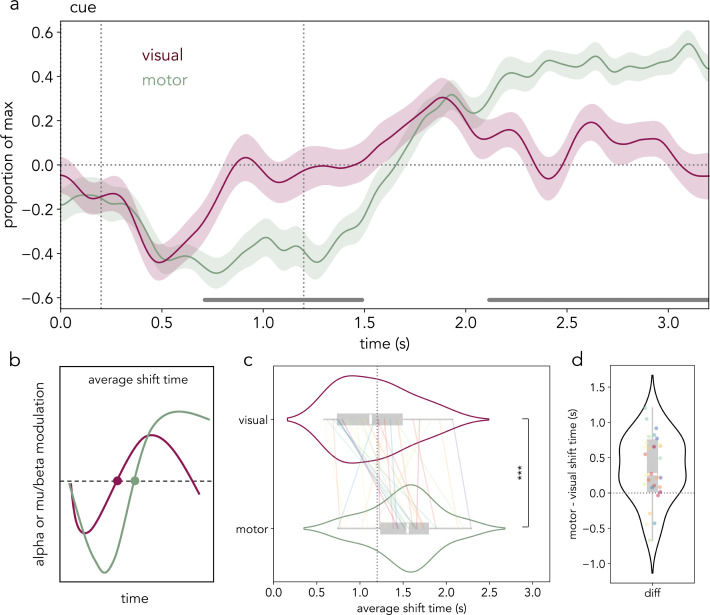
The lateralized alpha and mu/beta modulation time courses do not co-evolve in lockstep. **(a)** Average alpha (8−12 Hz) activity difference between contralateral and ipsilateral sensors to the cued location (burgundy) and average mu/beta (8−30 Hz) activity difference between contralateral and ipsilateral sensors to the cued action (green) scaled to range between −1 (minimum) and 1 (maximum) per participant (*N* = 30). Horizontal grey lines depict time points that were significantly different between the normalized alpha and mu/beta modulation time courses in a participant-wise cluster-based permutation test. Shaded areas represent the SEM. The vertical dotted lines represent (from left to right) the offset (0.2 s) of the retro-cue and the time of probe appearance in early trials (1.2 s). **(b)** Schematic representation of the average visual and motor shift time calculation procedure. For each participant, we calculated the minimum and maximum points of alpha and mu/beta modulation and estimated the average zero-crossing time (shift time; see “Materials and methods”). **(c)** Violin plots depicting the distribution of visual (top) and motor (bottom) average shift times across participants. Colored lines represent individual participant mean values, the white line inside the grey box represents the mean, and the edges of the grey box represent the first (left) and third (right) quartiles. Statistical significance is depicted with asterisks (***p < .001). **(d)** Violin plot depicting the difference between average motor and visual shift time for each participant (colored dots). The data in this figure can be found in OSF under *data/eeg/trf* [52]. The numerical values of the visual and motor shift times and their difference can be found in S3 Data.

Similar to what has been previously reported [23], visual inspection of lateralized alpha and mu/beta modulation showed they began largely together after the retro-cue (Fig 3A). Strikingly, the time courses of stimulus location and prospective hand action prioritization subsequently diverged into distinct trajectories. Specifically, the prioritization of location information seemed relatively short-lived in both early and late selection phases. Conversely, the prioritization of the first and then second prospective actions seemed longer-lasting. These differences suggest that, even when sensory and action-related contents co-exist in working memory, their relative prioritization can develop independently.

Thus, we investigated whether lateralized alpha and mu/beta activity modulations evolved in lockstep across the delay. First, we directly compared the time courses of sensory and motor prioritization in long trials, expressed as a proportion of their respective maximum and minimum. We found statistically significant differences between the normalized sensory and action prioritization time courses across participants (first cluster: ***p < .001; second cluster: ***p < .001; Fig 3A), which provided preliminary evidence that sensory and motor prioritization did not co-evolve in lockstep. This finding was supported by a linear mixed-effects model (LMM) of lateralized alpha and mu/beta modulation as a function of time and frequency band (alpha versus mu/beta) which revealed significant interactions between frequency band and time (S4 Fig and S5 Table).

Given the complementary roles of lower (8–12 Hz) and higher (13–30 Hz) mu/beta frequency activity in motor processes [60,61], we performed a supplementary analysis comparing alpha to lower and higher mu/beta modulations in sensorimotor sites relative to the relevant response hand. Lateralized modulations in both lower and higher mu/beta frequencies differed from the time course of the posterior alpha-frequency modulation associated with visual-spatial orienting to item location (S5 Fig).

Next, we investigated potential differences in the latency of the shift from prioritizing one location and action to the other, as indexed by the lateralized alpha and mu/beta modulation time courses, respectively. With this aim, the average time at which the alpha (visual) and mu/beta (motor) lateralization time series crossed the *y*-axis (i.e., reversed their lateralization) per participant was compared (Fig 3B; see “Materials and methods” for details). A paired-samples *t* test between the average *shift times* across participants revealed that the average *visual shift*, quantified on the lateralized alpha time series, occurred significantly earlier than the average *motor shift*, quantified on the lateralized mu/beta time series (*t*(*29*) = 4.2, ***p < .001, *d* = .79; Fig 3C and 3D; S3 Data). Interestingly, the average of the estimated *visual shift time* coincided approximately with the time of probe appearance in early trials (1.2 s from cue onset; M: 1.17 s; SD: .4 s). Alternatively, the *motor shift time* occurred at an average of 1.52 s (SD: .38 s) from cue onset, around 0.35 s (SD: 0.45 s) later than the visual shift time (Fig 3C).

**Fig 4:**
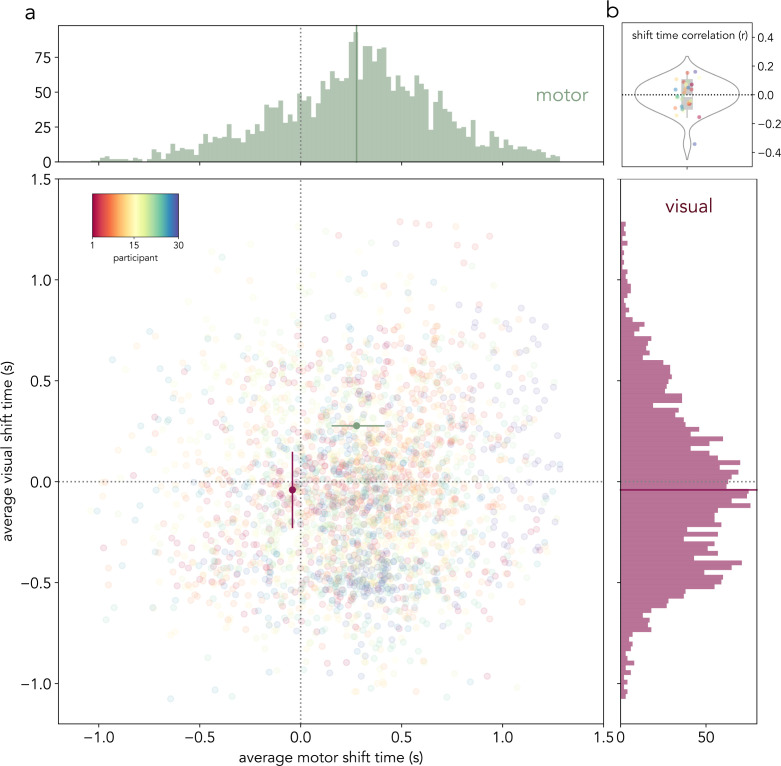
Lack of correlation between the shift times of the lateralized alpha and mu/beta modulation time courses. **(a)** Top and right: the histograms represent the distribution of average mu/beta (green) and alpha (burgundy) shift times, respectively, on all the iterations of the bootstrapping procedure (50 bins per histogram). Centre: scatter plot of the average alpha and mu/beta shift times in each of the 100 bootstrapping iterations across all 30 participants as plotted on the mu/beta (*x*-axis) and alpha (*y*-axis) time space. Both axes are centered around the time of early probe onset. Vertical and horizontal dotted lines represent the time of early probe appearance (1.2 s after retro-cue onset). The burgundy circle represents the participant-average visual shift time as estimated on the lateralized alpha modulation time course, and the green circle represents the motor shift time estimated on the lateralized mu/beta modulation time course. The lines on the left and bottom of the green and burgundy circles, respectively, depict the participant-average minimum motor and visual shift times. The lines on right and top the of the green and burgundy circles, respectively, depict the participant-averaged maximum motor and visual shift times. The colors of the scattered dots depict individual participants. **(b)** Pearson’s *r* values of the correlation between motor and visual average shift times across the 100 bootstrapping iterations for each participant (*N* = 30). Colored dots represent individual participants’ correlation values, the white line inside the grey box represents the mean, and the edges of the grey box represent the first (left) and third (right) quartiles. The data in this figure can be found in OSF under *data/eeg/trf* [52]. The numerical Pearson’s *r* values displayed in **(b)** can be found in S4 Data.

As further evidence that sensory and motor prioritization could evolve independently, we hypothesized that average sensory and motor shift times would be uncorrelated. Therefore, we investigated the cross-trial co-variability in average visual and motor shift timing using a bootstrapping procedure (see “Materials and methods”). Across 100 iterations per participant, the average motor shift time was not found to be a significant predictor of the average visual shift time in a LMM that included participant-related random effects (*β* = −.007, *t* = −.35, *p* = .72; Fig 4A). Next, we calculated the correlation (Pearson’s *r*) between the average visual and motor shift times across bootstrapping iterations per participant. A one-sample *t* test revealed that the participant-wise correlation values were not significantly different from zero (*t*(*29*) = −.37, *p* = .7, *d* = .1; Fig 4B; S4 Data). This lack of correlation between the average visual and motor shift times was replicated using other estimates of shift time, namely, the minimum visual and motor shift times and the minimum visual and motor inflection points (see S1 Methods and S6 Fig). The general absence of a correlation between visual and motor shift times further points to a functional decoupling of the lateralized alpha and mu/beta modulation time courses.

### Relation of lateralized alpha and mu/beta modulation to behavior

**Fig 5:**
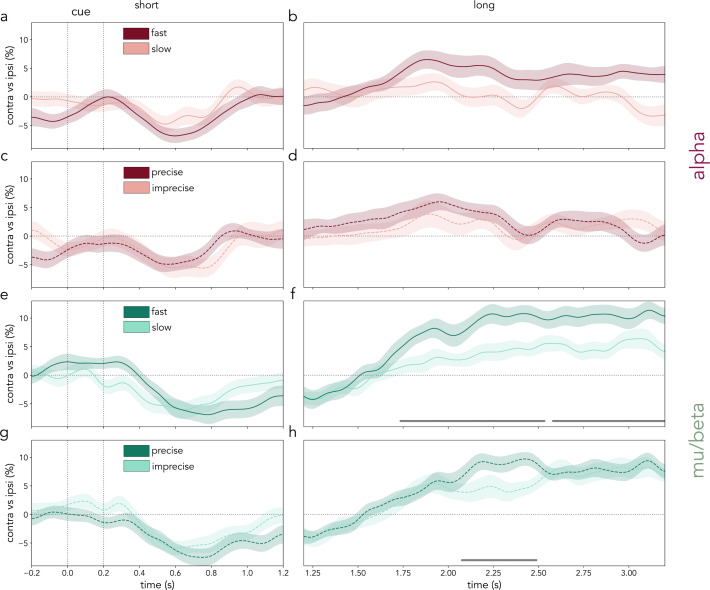
Relation between lateralized alpha and mu/beta modulations and behavior. Alpha and mu/beta activity contralateral vs. ipsilateral to the cued location/hand in fast vs. slow and precise vs. imprecise trials. **(a–d)** Average alpha (8–12 Hz; burgundy) activity difference between contralateral and ipsilateral occipital sensors to the cued location in trials categorized as fast (dark) or slow (light) based on a median split of RT **(a, b)** and in trials categorized as precise (dark) or imprecise (light) based on a median split of report error **(c, d)**. The left column depicts short trials **(a, c)** and the right column shows long trials **(b, d)**. **(e–h)** Average mu/beta (8–30 Hz; green) activity difference between contralateral and ipsilateral central sensors to the cued action in trials categorized as fast (dark) or slow (light) based on a median split of RT **(e, f)** and in trials categorized as precise (dark) or imprecise (light) based on a median split of report error **(g, h)**. The left column depicts short trials **(e, g)** and the right column shows long trials **(f, h)**. Cluster-based permutation significant time points of the contrast between the displayed time courses are indicated with horizontal grey lines (*N* = 30). Shaded areas represent the SEM. The data in this figure can be found in OSF under *data/eeg/trf* [52].

Next, the relation between the behavioral variables of interest (RT and report error) and the lateralized alpha- and mu/beta-frequency modulation patterns was investigated. To do so, trials were sorted based on a median split of performance (RT and error) per participant separately for short and long trials. Lateralized mu/beta activity modulations were found to relate to performance, as measured with both RT and report error. Long informative trials with faster responses also displayed a stronger modulation of mu/beta-frequency activity in the hemisphere contralateral to the hand of the second prospective action (first cluster: **p = .002; second cluster: ***p < .001; Fig 5F). Interestingly, despite not showing a general effect of informativeness on report error (Fig 1C), smaller errors (higher precision) in informative long trials were also related to a stronger lateralized mu/beta modulation (cluster: *p = .008; Fig 5H). No significant clusters in lateralized alpha activity were found in fast versus slow trials or precise versus imprecise trials (Fig 5A–5D), although a trend towards a stronger alpha modulation was observed in faster long trials (cluster: *p* = .08). Together, these results suggested that modulations in lateralized mu/beta activity were tightly related to performance, in particular the modulations that signaled the change of action plan as time elapsed.

### Oscillatory dynamics underlying the lateralized alpha and mu/beta contrasts

**Fig 6:**
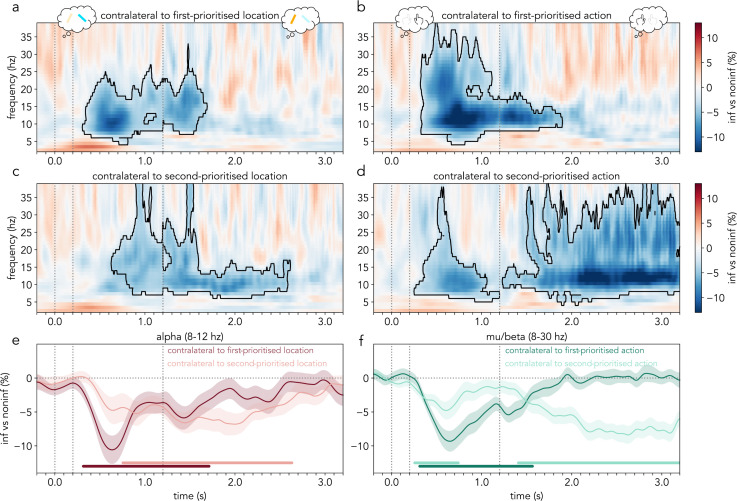
Time–frequency activity difference in informative vs. noninformative (neutral) trials in sensors contralateral to the first- and second-prioritized locations and actions. **(a, c)** Difference in time–frequency activity in occipital (PO7/PO8) channels contralateral to the first-prioritized location **(a)** and second-prioritized location **(c)** in informative trials vs. noninformative trials (expressed as a percentage). **(b, d)** Difference in time–frequency activity in central (C3/C4) channels contralateral to the first-prioritized action **(b)** and second-prioritized action **(d)** in informative trials vs. noninformative trials (expressed as a percentage). **(e)** Cross-participant average alpha (8−12 Hz) activity difference between informative and noninformative trials (%). Burgundy: channels contralateral to the first-prioritized location. Pink: channels contralateral to second-prioritized location. **(f)** Cross-participant average mu/beta (8−30 Hz) activity difference between informative and noninformative trials (%). Dark green: channels contralateral to the first-prioritized action. Light green: channels contralateral to second-prioritized action. Black outline in time–frequency spectra indicates statistically significant clusters **(a**–**d)**. Shaded areas represent the SEM and cluster-permutation corrected significant time points are indicated with horizontal lines in **e** and **f** (*N* = 30). The first part of the time–frequency spectra in panels **a–d** and of the time course in **e** and **f** (−0.2–1.2 s) corresponds to the average of short and long trials, and the second part (1.2–3.2 s) corresponds to long trials only. The vertical dotted lines represent (from left to right) the onset (0 s) and offset (0.2 s) of the retro-cue and the time of probe appearance in early trials (1.2 s). The data in this figure can be found in OSF under *data/eeg/trf* [52].

Lateralized alpha and mu/beta activity modulations are meaningful markers of the flexible and dynamic prioritization of sensory and action-related working-memory contents. They are typically quantified as compound markers, combining potential contributions of contralateral power decreases related to increases in cortical excitability and ipsilateral power increases related to decreases in cortical excitability [24,25,26,34–38]. To explore the dynamics of putative increases and decreases in cortical excitability, we utilized noninformative (50%) trials – in which, arguably, no specific location or action was prioritized until the response probe – as a “neutral” baseline. Specifically, we compared time–frequency activity in contralateral and ipsilateral occipital (“visual”) and central (“motor”) channels with time–frequency activity in the same channels during noninformative (neutral) trials (Fig 6).

Across the board, we found desynchronizations of oscillatory activity in informative relative to noninformative (neutral) trials, which were particularly pronounced in the alpha and mu/beta frequency bands (8–30 Hz; Fig 6). Alpha-frequency power in occipital (“visual”) channels contralateral to the first-prioritized location was attenuated relative to the neutral baseline after informative retro-cues. The effect started during the first delay and persisted into part of the second delay in long trials (cluster: ***p < .001; Fig 6A). Alpha power in occipital channels contralateral to the second-prioritized location also decreased. The effect started later during the first delay and persisted longer into the second delay in long trials (cluster: ***p < .001; Fig 6C).

Similarly, mu/beta-frequency activity in central (“motor”) channels contralateral to the first-prioritized action decreased relative to neutral-cue trials. The effect started early during the first delay and persisted into part of the second delay (cluster: ***p < .001; Fig 6B). Mu/beta-activity contralateral to the second-prioritized action was also attenuated. Interestingly, two separate clusters were observed – an early cluster during the first delay (first cluster: *p = .02; Fig 6D) and a second cluster lasting for most of the second delay (second cluster: ***p < .001; Fig 6D). The average time courses of alpha (8–12 Hz) and mu/beta (8–30 Hz) activity relative to the neutral baseline in channels contralateral to the first- and second-prioritized attributes (locations and actions, respectively) confirmed the above pattern of findings (Fig 6E and 6F; E: first cluster: ***p < .001; second cluster: ***p < .001; F: first cluster: *p = .04; second cluster: ***p < .001).

These findings additionally reveal that the contra-vs-ipsi power changes related to location and action prioritization (Fig 2) emerge from spatiotemporally distinct patterns of oscillatory desynchronization. They show that, in our data, these modulations are driven predominantly by contralateral decreases in spectral power rather than ipsilateral increases in spectral power, relative to our neutral condition with noninformative retro-cues. Interestingly, while the relative desynchronization time series contralateral to the first-prioritized location and action follow similar time-courses (darker lines in Fig 6, panels E and F), the desynchronization profiles related to the second-prioritized visual and motor attributes are notably different from each other (lighter lines in Fig 6E and 6F). This suggests that the reported decoupling between the lateralized alpha and mu/beta modulation time-courses depicted in Fig 3 may stem predominantly from differences in the prioritization of the second-selected location and action.

## Discussion

The present study reveals that both sensory and action-related contents that co-exist in working memory are flexibly and dynamically prioritized in a temporally structured but independent fashion.

In this task, both retro-cues and internal states related to changing temporal expectations successfully elicited attentional prioritization of stimulus locations and associated actions in working memory. Across two sessions, response times were faster when responding to targets at their expected times. Importantly, the speed of responses to previously un-prioritized items was comparable to first-prioritized item responses, highlighting the flexibility and reversibility of internal attention (see [9,40–43,45,46,48,62]). Perhaps due to participant over-training, accuracy was exceptionally high in this task and insensitive to attentional selection. Reaction times were faster in informative and noninformative long trials versus short trials, possibly reflecting the larger conditional probability of an item being probed (50% in short, 100% in long) and/or increased preparedness to respond following a longer delay [53]. While no effects of informativeness were identified on report error, longer trials had higher errors in both informative and noninformative conditions, possibly due to a time-related degradation of working-memory contents [63] or because of a speed-accuracy trade-off.

Mirroring the behavioral results, the lateralized alpha and mu/beta modulation time courses uncovered that the prioritization of both sensory- and action-related contents in working memory was flexible (i.e., it “shifted” from prioritizing the first to the second location/action) and temporally tuned (i.e., both instances of selection of the relevant item/action were temporally specific).

The pattern of lateralized sensory (alpha) modulation in the present study replicates previous findings that the prioritization of sensory contents in working memory is flexible [9,40–43,46,48,62,45] and temporally tuned [32,51]. Consistent with previous studies, the short-lived modulation of lateralized alpha activity in the first and second selection events points to a transient relative prioritization of item locations (see also [28,31,40,41,64,65]). This has been suggested to reflect that spatial orienting helps “activate” working-memory contents into a prioritized state [1]. In line with previous reports (e.g., [28,54–57]), spatial selection in this task occurred even though the locations of items were not strictly necessary for task performance. Despite the irrelevance of spatial information, location-related lateralized alpha modulation was seen not only in the first instance of location selection, but also when location prioritization was endogenously driven by temporal expectations. In contrast to other studies (e.g., [30]), here, lateralized alpha-frequency activity during the memory delay was not systematically correlated with behavior. We speculate that this could be related to the absence of a behavioral effect of selection on report error and the smaller magnitude of lateralized alpha modulation compared to mu/beta in this study.

To the best of our knowledge, the present study is the first to reveal the flexible, reversible, dynamic, and temporally tuned nature of proactive action-related prioritization in working memory. Specifically, action-related contents that were relatively deprioritized in the first instance could be re-prioritized later as indexed by a strong mu/beta modulation and behavioral benefits in long trials. Lateralized mu/beta modulation persisted well into the second part of the delay, after the time of early probe appearance had passed. This accords with the requirement to respond using the selected effector (left or right hand) at the end of the first or second selection period.

Importantly, the extent of lateralized mu/beta modulation in the second memory delay correlated with enhanced task performance. Lateralized mu/beta modulation was enhanced in faster trials as opposed to slower ones (see also [14,19]), suggesting that the RT effects in this task may be reflective of differences in motor preparation. Interestingly, the relation between lateralized mu/beta modulation and behavior only reached statistical significance during the latter part of the delay, when only one certain prospective action was held in working memory. This poses questions about whether certain and immediate prospective actions modulate lateralized oscillatory activity differently from uncertain actions (but see [19,20]) or subsequent goal-relevant items (see also [40,41,66]). Moreover, despite the insensitivity of report error to cue informativeness, lateralized mu/beta-frequency activity during the second part of the memory delay was stronger in precise than imprecise trials, highlighting the importance of motor states in internal attention. Together, these findings point to a key role for lateralized mu/beta activity in flexibly prioritizing motor contents in working memory and highlight its value as a functionally relevant marker of internal attention.

A central finding of the current study with important theoretical consequences was the temporally dissociable modulation of sensory and action-related contents in working memory, as measured by the lateralized modulation of alpha and mu/beta activity. Their flexible and dynamic prioritization progressed in tandem but was uncoupled. In the first moments after the retro-cue, sensory and action-related prioritization started together, consistent with previous findings [23,67]. However, in contrast to the transient nature of lateralized alpha modulation in this task, lateralized mu/beta activity showed a longer-lasting motor modulation. The present findings are consistent with previous studies suggesting that working-memory contents may undergo a change from a sensory to a motor code as the requirement to respond takes precedence in the task [1,16–18,33]. Despite potential differences in the role of lower (8–12 Hz) and higher (13–30 Hz) frequencies of mu/beta activity in motor processes [60,61], in the present study, both frequency ranges were similarly decoupled from location-related alpha modulation.

Crucially, as expectations about the to-be-probed memory representation reversed, the lateralized sensory and action-related modulation signals evolved independently. The relative modulation of sensory and motor systems by internal attention, as measured with lateralized alpha and mu/beta activity modulations, did not co-evolve in lockstep. Even though they both showed a clear reversal with time, this reversal developed distinctly for both markers and its exact timing was uncorrelated. Together, these findings suggest that sensory and action-related dimensions of working-memory representations need not be intrinsically bound but instead can be dissociable and susceptible to separate modulatory processes. In turn, this highlights the multiplicity of modulatory processes that can operate on working-memory representations contemporaneously but independently [3,6].

Here, the lateralized modulation of location-related activity was transient, while the lateralized modulation of motor activity was longer-lasting. The patterns match the changes in when and for how long location- and action-related information was relevant to this task. The response-hand continued to be relevant until the end of the trial, while location information was not strictly necessary for the task and may have become superfluous following item selection. This is in line with several other findings highlighting the short-term dynamics of lateralized alpha modulation during visual attention and working memory (e.g., [28,40,41,65,68]). The transient nature of lateralized alpha modulation in this task may have contributed to the variability of visual shift times observed in this study. More generally, we speculate that different attributes of working memory objects may be differentially modulated depending on changing expectations about their relevance. Relatedly, external attention has also been shown to differentially modulate spatial-sensory and motor systems when the purpose of the task emphasizes perceptual demands versus speeded responses, respectively [69].

We measured lateralized alpha and mu/beta activity modulations to track relative changes in local neural excitability in visual and motor regions related to internal selective attention. These signals could reflect multiple underlying neural mechanisms, which remain debated and investigated [34–37,70]. For example, the transient modulation of lateralized alpha followed by its quick return to baseline could reflect both a passive de-prioritization of the cued location and/or the active deallocation of attention from that location.

Interestingly, the present study revealed that the modulation of alpha and mu/beta activity related to location and action prioritization, respectively, primarily reflected attenuation of oscillatory power. Alpha and mu/beta power initially decreased over the scalp contralateral to the cued location and response hand and then decreased over the scalp contralateral to the second-prioritized attributes in long trials. The pattern of modulation contrasts with the proposal that alpha modulation primarily reflects gains in ipsilateral power resulting in distractor inhibition [37]. Intriguingly, oscillatory activity contralateral to the second-prioritized location and action was modulated from relatively early after the retro-cue. To the extent that the observed modulations reflect enhanced processing of the second-prioritized location and action, they are in line with previous studies highlighting early and prospective prioritization of location- and action-related contents [14,19,47]. Specifically, the present results echo earlier reports of early prioritization of future-relevant sensory attributes, even when competing sensory representations are currently relevant [47] and prospective action planning at early stages of sensory processing [14,19]. Together, these findings add to a growing body of research highlighting the multiplexing of working-memory contents by internal attention.

In summary, the present study shows that sensory and action-related contents that co-exist in working memory are flexibly and dynamically prioritized and tuned to the moments when they are anticipated to become relevant. This points to a fine temporal organization of internal attention according to external temporal regularities. Moreover, the prioritization of sensory and action-related contents that co-exist in working memory was not continuously temporally coupled. This speaks to the relative independence of object dimensions in working memory and the multiplicity of flexible and dynamic modulatory processes that co-occur to prepare internal representations for adaptive behavior.

## Materials and methods

### Participants

This study was approved by the Central University Research Ethics Committee of the University of Oxford (R57489/RE006). This study was conducted according to the principles expressed in the Declaration of Helsinki. The sample size of the present study was based on previous studies investigating related questions [14,23]. Thirty-one volunteers participated in two experimental sessions, which took place on different days. All participants self-reported having normal or corrected eyesight. They provided written consent before each session and were reimbursed at £15/h for their participation.

Exclusion criteria were pre-defined. Participants were excluded from further analyses if their average performance error or reaction time (RT) was above 3 standard deviations (SDs) from the mean error or RT, respectively, across all participants in either session. Data from one participant were excluded from further analyses based on these criteria. The final sample (*n* = 30) had an average age of 23.57 (SD: 3.85). Three individuals were left-handed and 27 were right-handed by self-report; six participants identified as male, 23 as female, and one as non-binary.

### Experimental procedure and stimuli

We designed a visual-motor working-memory task to study the flexible and dynamic prioritization of visual and action-related contents in working memory (Fig 1A). Participants were shown two colored, tilted bars and were asked to report the tilt of one of the bars at the end of each trial. Retro-cues that matched or did not match the color of one of the encoded bars were informative or noninformative regarding the item participants had to report at the end of the trial, respectively. Participants reported the orientation of one of the encoded bars at the end of each trial. Importantly, bar orientation (left versus right) was linked to the response hand (left versus right). Additionally, the location of the bars on the screen (left versus right) and the direction of their orientation (left versus right) were manipulated orthogonally. Therefore, the prioritization of bar locations and prospective hand actions could be tracked independently using their respective EEG markers (see also [14,19,23,58]). Specifically, we used lateralized alpha (8–12 Hz) activity modulation in occipital channels as a proxy of spatial prioritization (e.g., [23,28–33]) and lateralized mu/beta (8–30 Hz) activity modulation in central channels as an index of action preparation (e.g., [22–24]). Given the orthogonal manipulation of bar location and response hand, the trial-average spatial alpha and mu/beta modulations (as a function of item location and required response hand) could be independently quantified.

The experimental script was generated using the Psychophysics Toolbox version 3.0.18 [71] on Matlab 2022a [72]. Participants sat in a dimly lit room, approximately 60 cm away from a monitor (Dell U2312HM; 1920 × 1080 pixels resolution; 100-Hz refresh rate).

The display background was grey (#7F7F7F) for the duration of the task. At the beginning of each trial, participants saw the encoding display made of a white central fixation cross (#FFFFFF; 14 pixels) and two colored, tilted bars shown for 250 ms. One bar was always on the left and the other on the right side of the screen. Each bar was centered 192 pixels (5.2 DVA) away from the central fixation cross, had a length of 192 pixels and a width of 38 pixels. One bar was always tilted leftward and the other rightward. Bar tilts were drawn from one of three possible bins with a uniform distribution of orientations (±10–33°, ±34–57°, and ±58–80°). To avoid cardinal effects, none of the displayed bars had orientations along the vertical or horizontal meridians. Orientation bins were counterbalanced so that all bins were equiprobably sampled for each participant. Bar location and tilt were orthogonally manipulated, such that leftward- and rightward-tilted bars were equally likely to appear on the left or right side of the screen across trials. Importantly, bar tilt (leftward versus rightward) was directly related to the hand (left versus right) with which participants would be asked to respond at the end of the trial. The two bars on the encoding display had two different colors out of four possible, highly distinguishable colors: light blue (#00DEFF), orange (#FFAC00), pink (#FF62FF), and green (#00ED82). Colors were counterbalanced across trials such that all possible combinations of color, location, and tilt were displayed in each block.

Following the encoding display, the colored bars disappeared, and the central fixation cross remained on the screen for 750 ms (first delay; Fig 1A). Subsequently, the color of the central fixation cross changed for 200 ms (retro-cue). In half of the trials (*informative;* 50%), the color of the retro-cue matched the color of one of the two bars displayed previously. In the other half of the trials (*noninformative;* 50%), the fixation cross changed to a different color that matched neither encoded item on that trial. The cue color was chosen from the four possible colors detailed above. Cue color was counterbalanced such that all colors acted as cues in the same number of trials across each block of the task and appeared equiprobably in informative and noninformative trials.

In half of the informative and half of the noninformative trials, the response probe appeared 1 s after cue offset (*short* trials; 50%). In the other half of the trials, the response probe appeared 3 s after cue offset (*long* trials; 50%). During the delay (short and long), only a white, central fixation cross remained on the screen.

Crucially, in informative trials, the combination of two pieces of information predicted which of the two bars would be probed at the end of the trial with 100% validity: (1) the color of the retro-cue and (2) the duration of the delay between the cue and the probe. When the cue was informative (matching the color of one of the two bars displayed at encoding) and the delay following the cue was short (1 s), participants were prompted to report the orientation of the cued bar at the end of the trial (100% validity). Alternatively, in informative trials with a long delay (3 s), participants were always required to report the orientation of the other (uncued) bar (100% validity). Therefore, participants were encouraged to attend to the retro-cue and to track the duration of the subsequent delay to know which of the two bars they would be asked to report. In informative trials, they could anticipate reporting the item with the cued color after the short interval. Once the short interval passed, they could shift the focus of attention to the item with the other color. In noninformative trials, which of the two bars would be probed was unpredictable to participants. Informative and noninformative trials were randomly interspersed in this task.

Following the delay, the color of the central fixation cross changed again (probe) prompting participants to reproduce the tilt of the color-matching bar (Fig 1A). To this end, participants used the “F” and “J” keys on the keyboard with their left and right index fingers respectively. Upon response initiation, a grey bar appeared centrally in the vertical position. Pressing the “F” key caused a counterclockwise rotation of the grey bar and pressing the “J” key caused a clockwise rotation. While the button was being pressed, the bar moved at a speed of 1 degree every 8 ms, thus requiring 820 ms to bring the dial from vertical (0°) to horizontal (90°). The grey bar did not rotate further than 90° in either direction (see also [23]). The response key could not be changed once a response was initiated. Therefore, leftward tilted bars could only be accurately reported with the left hand, and rightward tilted bars with the right hand. Participants confirmed their response by releasing the key when the grey bar reached the desired orientation. The size of the grey bar was the same as that of the colored bars on the encoding display. Participants were instructed to report the orientation as quickly and accurately as possible. If participants had not completed their response within 3 s after probe onset, the next trial began.

If participants responded with the correct key (F for leftward-tilted bars and J for rightward-tilted bars), they received visual feedback about the percentage accuracy of their response. The percentage accuracy reflected the accuracy of their report with a 0° error leading to a “100%” feedback and 90° error reflecting “0%”. If they pressed the incorrect key, they received the message “Wrong target!”. If they did not respond, they saw “Too slow!”. All feedback appeared centrally in white letters and was displayed for 500 ms. Inter-trial intervals (ITIs) followed a beta distribution with a minimum of 3 s, maximum of 10 s, and average of 5.5 s. During each ITI, a white fixation cross was displayed centrally.

The present study was divided into two separate sessions in which participants repeated the same task procedure on different days. The two visits allowed participants to become familiar with the task instructions and procedures. The first visit consisted of a behavioral session in which participants completed eight blocks of 32 trials each for approximately 1 h. The second visit consisted of an EEG and eye-tracking session in which participants completed 15 blocks with 32 trials each for approximately 2.5 h. In the EEG session, participants could rest for 10–15 min after every 5 blocks. In each block, there was an equal number of informative and noninformative trials. Before beginning the first session of the experiment, participants were walked through the task instructions and asked to practice the procedure for at least one block.

### Behavioral data analysis

Behavioral data were analyzed using the R statistical programming language (version 4.2.1; [73]) and Rstudio (version RStudio 2022.07.1; [74]). Report errors were calculated as the absolute difference between the reported orientation and the orientation of the probed bar. RT was defined as the time from probe onset until response initiation. Trials without a response, trials with RTs faster than 100 ms, trials with RTs slower than three times the SD of the average RT per participant, and trials with report errors higher than three times the SD of the participant-averaged error were excluded from further behavioral analyses. This resulted in an average removal of 2.38% (SD: 0.72%) of trials in the behavioral session and 2.82% (SD: 0.6%) in the EEG session. The average RT and report error per participant and per condition were calculated for each session. Given the equivalent behavioral pattern across sessions (S1–S4 Tables), RT and report errors from both sessions were collapsed (Fig 1B and 1C). Additionally, we calculated the average response density as a function of the reported tilt and the tilt of the probed item (Fig 1D).

The statistical significance of the dependent variables of interest across participants (report error and RT) was tested using 2 × 2 repeated-measures analysis of variance (ANOVA) with informativeness (informative and noninformative) and duration (short and long) as factors. The metric of effect size was *η*^2^ and the within-subject standard error of the mean (SEM) was quantified using the normalized data [75].

### EEG: acquisition and preprocessing

EEG was acquired using a 64-channel Quik-Cap Neo Net cap (Ag/AgCl electrodes), Synamps amplifiers, and the CURRY 8 acquisition software (Compumedics Neuroscan). Sixty-four channels were distributed across the scalp following the international 10−10 positioning system. Data were referenced online to a reference channel positioned between Cz and CPz. Another channel (Afz) was used as the ground. Vertical and horizontal electrooculograms (EOG) were simultaneously recorded using a bipolar system integrated into the cap. Horizontal EOG electrodes were placed on the side of each eye and vertical EOG was positioned above and below the left eye. The electrocardiogram (ECG) was measured with an integrated, bipolar set-up. The upper ECG was placed on the left ribcage, centered above the left chest; the lower ECG was placed on the side of the left ribcage. During set-up, electrode impedance was lowered to below 5 kΩ where possible and was at least below 10 kΩ. Data were digitized at 1,000 Hz and filtered at 500 Hz during acquisition.

All EEG data were processed and analyzed in Python 3.11.9 [76] using MNE-Python (version 1.5.1; [77]) and custom-made scripts. First, data were notch-filtered at 50 Hz, and high-pass (0.05 Hz) and low-pass (40 Hz) filtered with a Finite Impulse Response (FIR) filter. Subsequently, the continuous data were visually inspected. Channels deemed “bad” were interpolated using spline interpolation as implemented with the *interpolate_bads* function in MNE-Python. Then, data were downsampled to 250 Hz and re-referenced to the sensor average. An independent component analysis (ICA) identified artifacts related to eye movements and heart-related activity by correlating individual ICs with the EOG and ECG signals. Based on the correlation values and visual inspection of the IC time courses and topographies, ICs capturing eye- and heart-related activity were identified and subtracted from the EEG data. An average of 2.87 ICs (SD: 0.8; range: 1–5) were removed per participant.

Subsequently, the raw EEG data were epoched around cue onset and probe appearance (−0.25 to 1.25 s in short trials and −0.25 to 3.25 s in long trials), and activity during the baseline period (−0.25 to 0 s) was subtracted from each epoch. A surface Laplacian transform was applied to the epoched data using the *compute_current_source_density* MNE-Python function to reduce the effects of volume conduction and thus increase the spatial resolution and interpretability of the results (see also [23]).

Trials included in the EEG analysis were limited to those in which participants had pressed the correct key and RT was higher than 100 ms. Noisy trials, as identified on the epoched EEG time courses using a Generalized ESD procedure [78], were also excluded. In total, 89.67% (SD: 5.34%) of trials were used in subsequent analyses.

### EEG: time–frequency analyses

All EEG analyses focused on the period of interest from retro-cue onset until probe onset (see Fig 1A). The retro-cue in informative trials was hypothesized to prompt participants to direct attention to the cued sensory (location) and action-related (response hand) contents in working memory. Importantly, it was hypothesized that in long trials, participants would “shift” to prioritizing the other stimulus location and response hand after the short interval lapsed. Crucially, given the orthogonal manipulation of bar location and tilt, prioritization of locations and action plans could be tracked independently with EEG (see also [14,19,23]). Specifically, it was hypothesized that EEG alpha-frequency activity (8–12 Hz) at occipital electrodes contralateral-vs-ipsilateral to the location of the prioritized item would be modulated. In parallel, it was predicted that EEG mu/beta activity (8–30 Hz) at central electrodes contralateral-vs-ipsilateral to the prioritized response hand would be modulated. Alpha-band activity (8–12 Hz) at left (PO7) and right (PO8) visual sensors was used to track sensory-related prioritization. Mu/beta activity (8–30 Hz) in left (C3) and right (C4) motor electrodes was used to track action-related prioritization (see also [21,33]). For confirmation purposes, the same analyses were performed using a larger cluster of lateralized visual and motor sensors (S3 Fig).

The epoched EEG time series were transformed into their time–frequency decompositions using the SAILS Python toolbox [79,80]. This was done by convolving the time series with a complex 3-cycle Morlet wavelet from 2 to 40 Hz in steps of 1 Hz. The 50 ms around the two edges of each epoch were subsequently cropped to remove any edge artefacts related to the time–frequency decomposition. Time–frequency activity in lateralized visual (PO7/PO8) and motor (C3/C4) channels was contrasted between trials in which either the stimulus location or the prospective hand action (related to the tilt), respectively, was contralateral versus ipsilateral to each channel. This was calculated and normalized as follows: [((contra − ipsi)/(contra + ipsi)) * 100], separately for left and right sensors per participant. Subsequently, the contrast across both sides was collapsed in the visual and motor selection conditions separately and averaged across participants.

The same procedure was followed with each symmetrical electrode pair to create the topographical lateralization maps shown in Fig 2. The topographies depict the contra-vs-ipsi contrasts at the time points corresponding to the statistically significant clusters in the panels above (Fig 2A and 2B).

A similar procedure was followed to calculate the difference between time–frequency activity in informative trials versus noninformative trials: [((inf − noninf)/(inf + noninf)) * 100]. This was estimated separately for channels contralateral to the first-selected location/action and channels contralateral to the second-selected location/action.

To generate the time course of posterior alpha and central mu/beta modulations, signals from the pre-defined frequencies (alpha: 8–12 Hz; mu/beta: 8–30 Hz) were averaged at the selected channels (Figs 2C, 2D, 6E, and 6F). To increase sensitivity and improve visualization, the trial-averaged alpha and mu/beta time courses for each participant were smoothed using a Gaussian kernel with a standard deviation of 40 ms (see also [23]).

To increase their comparability, the lateralized alpha and mu/beta modulation time courses were each scaled to range between −1 and 1 per participant. Subsequently, we used cluster-based permutation (see below) to statistically compare the alpha and mu/beta time courses across participants (Fig 3A). The average time–frequency activity in the early period (−0.2 to 1.2) includes both short and long trials. The later period (1.2 to 3.2) includes only long trials.

### EEG: average visual and motor shift time quantification

Next, the temporal relation between the time courses of the two frequency bands of interest (alpha and mu/beta) was investigated. With this aim, a set of key control points was identified on the trial-average time courses. First, the participant-averaged lateralized alpha and mu/beta time courses were smoothed using a Gaussian kernel with a standard deviation of 120 ms to facilitate the identification of the control points. To enhance comparability between the signals, the lateralized alpha and mu/beta time courses were scaled to range between −1 and 1, respectively. For each participant, the minimum point of lateralized alpha and mu/beta modulation was identified within a pre-defined time window (0.1 to 1 s from cue onset), and the maximum point of lateralized alpha and mu/beta modulation was identified in a later window (1 to 2.5 s from cue onset). Subsequently, the average time of zero crossing (i.e., lateralization reversal) between the identified minimum and maximum points was identified as the average shift time, as depicted in Fig 3B. When participants had multiple zero crossings, the average shift time was calculated. When participants had a single zero crossing, we considered the latter to be the average shift time. A two-sided paired-samples *t* test compared the average shift times of the alpha and the mu/beta time courses across participants (Fig 3C). The effect size was reported as Cohen’s *d*.

Next, we followed a bootstrapping procedure in which half of the trials in each participant were randomly sub-sampled over 100 iterations (Fig 4A). For every subset of trials from a single participant, the contralateral-versus-ipsilateral alpha and mu/beta modulation time courses were calculated, smoothed, and scaled as detailed above. Subsequently, the control points described above were identified. Next, we tested for any existing correlations between the average visual shift time and the average motor shift time across all iterations using a linear mixed-effects model (LMM; using the *lmer* from the R package *lme4*;  [81]) that incorporated participant-related random effects as follows:


mean visual shift~mean shift time+(1 | participant)

The model was estimated using a maximum likelihood criterion, and the outputs of the model were reported as unstandardized regression coefficients with *t*-statistics. We used two-tailed tests and a 5% criterion for significance.

Finally, we calculated the correlation (Pearson’s *r*) between the average visual and motor shift times across all bootstrapping iterations per participant. The resulting *r* values were statistically compared against a null hypothesis of 0 with a one-sample *t* test (Fig 4B).

### EEG: relation to behavior

We investigated the relation between the two behavioral dependent variables of interest (report error and RT) and the lateralized modulation of alpha- and mu/beta-frequency activity during the delay in informative trials separately for trials with a long and a short delay. For each participant and trial type (short and long), the median RT was estimated, and the trials were divided into those that were faster (fast) and those that were slower (slow) than the median RT. In parallel, the median report error was calculated. Trials were then separated into those with more (precise) or less accurate (imprecise) reports than the median error. Subsequently, we calculated and compared the contralateral-versus-ipsilateral alpha and mu/beta contrasts in fast versus slow trials and in precise versus imprecise trials (Fig 5).

### Cluster-based permutation testing

Cluster-based permutation [82] tested for statistical differences in the contralateral-versus-ipsilateral time–frequency spectra in informative versus noninformative trials, the alpha and mu/beta modulation time courses and the informative versus noninformative time–frequency spectra contrasts. This statistical approach assumes the effects of interest are clustered across the relevant dimensions (e.g., time, time–frequency, space–time–frequency), making it suitable for testing the statistical significance of EEG activity patterns.

First, a mass-univariate *t* test (two-sided, alpha = .05) was performed on the group-level contrasts, and the sum of all *t*-values in a cluster was defined as the cluster statistic for each given cluster. Next, the contrasts across the conditions of interest were randomly permuted (sign-flipped) 10,000 times for each participant. The largest clusters found under this null hypothesis were compared with the cluster statistics in the observed data. For each cluster, the proportion of permutations whose largest cluster exceeded the cluster identified in the observed data was calculated and the resulting *p*-value was estimated. Importantly, this approach circumvents the common problem of multiple comparisons by comparing distributions of the summary cluster statistics.

## Supporting information

S1 Fig Lateralized, frequency-specific EEG activity locked to cue onset in noninformative trials, where no location/action was systematically prioritized. **(a)** Contrast between EEG time–frequency activity contralateral versus ipsilateral to the cued bar location (none) in occipital sensors (PO7, PO8) divided by summed contralateral and ipsilateral activity and expressed as a percentage in noninformative trials. **(b)** Contrast between EEG time–frequency activity contralateral versus ipsilateral to the cued prospective action (none) in central sensors (C3, C4) divided by summed contralateral and ipsilateral activity and expressed as a percentage in noninformative trials. The first part of the time–frequency spectra (−0.2–1.2 s) corresponds to the average of short and long trials, and the second part (1.2–3.2 s) corresponds to long trials only. The vertical dotted lines represent (from left to right) the onset (0 s) and offset (0.2 s) of the noninformative cue and the time of probe appearance in early trials (1.2 s). No significant clusters were found (*N* = 30). For comparison purposes, the time–frequency spectra are plotted on the same scale as Fig 2A and 2B. The data in this figure can be found in OSF under *data/eeg/trf* [52].
(PDF)

S2 Fig Horizontal gaze position as a function of cued item location locked to retro-cue onset in informative trials. **(a)** Participant-averaged horizontal gaze position (in pixels) when an item on the left (dotted line) vs on the right (solid line) was cued locked to cue onset in informative trials (first cluster: *p = .01; second cluster: ***p < .001). **(b)** Participant-averaged towardness (metric which collapses across left and right cued items) locked to cue onset in informative trials (black; first cluster: **p = .009; second cluster: *p = .02). Shaded areas represent the SEM, and vertical dotted lines represent (from left to right) cue offset and time of probe appearance in early trials. Cluster-permutation significant time points are indicated with horizontal lines at the bottom of the plots (*N* = 30). The first part of the time courses (0–1.2 s) is the average across both short and long trials, and the second part (1.2–3.2) averages across long trials only. The data in this figure can be found in OSF under *data/eye* [52].
(PDF)

S3 Fig Lateralized, frequency-specific EEG activity locked to cue onset in informative trials in a cluster of visual/motor EEG electrodes. **(a)** Contrast between EEG time–frequency activity contralateral versus ipsilateral to the cued bar location in two clusters of lateralized occipital sensors (L: O1, PO7, PO3; R: O2, PO8, PO4) divided by summed contralateral and ipsilateral activity (expressed as a percentage) in informative versus noninformative trials. Black outline indicates significant clusters. **(b)** Contrast between EEG time–frequency activity contralateral versus ipsilateral to the cued prospective action in two clusters of lateralized central sensors (L: C1, C3, CP1, CP3; R: C2, C4, CP2, CP4) divided by summed contralateral and ipsilateral activity (expressed as a percentage) in informative versus noninformative trials. Black outline indicates statistically significant clusters. **(c)** Average alpha (8–12 Hz) activity difference between contralateral and ipsilateral sensors to the cued location across participants (burgundy) in informative trials. Average mu/beta (8–30 Hz) activity between contralateral and ipsilateral sensors to the cued action across participants (green) in informative trials. Shaded areas represent the SEM and cluster-based permutation-corrected significant time points are indicated with horizontal lines (burgundy: alpha versus null; green: mu/beta versus null; grey: alpha versus mu/beta; *N* = 30). The first part of the time–frequency spectra in panels **a** and **b** and of the time course in **c** (−0.2–1.2 s) corresponds to the average of short and long trials, and the second part (1.2–3.2 s) corresponds to long trials only. The vertical dotted lines represent (from left to right) the onset (0 s) and offset (0.2 s) of the retro-cue and the time of probe appearance in early trials (1.2 s). For comparison purposes, the time–frequency spectra are plotted on the same scale as Fig 2. The data in this figure can be found in OSF under *data/eeg/trf* [52].
(PDF)

S4 Fig Linear mixed-effect model of the lateralized alpha and mu/beta modulation time courses as a function of time. Average alpha (8−12 Hz) activity difference between contralateral and ipsilateral sensors to the cued location in informative long trials (burgundy) and average mu/beta (8−30 Hz) activity between contralateral and ipsilateral sensors to the cued action (green) in informative long trials, both scaled to range between −1 and 1 and centered around the time of early probe onset (1.2 s). The dark lines depict the lateralized alpha and mu/beta modulation values as estimated with the linear mixed-effects model (see S1 Methods). Shaded areas represent the SEM (*N* = 30). The vertical dotted lines represent (from left to right) the offset (−1 s) of the retro-cue and the time of probe appearance in early trials (0 s). The data in this figure can be found in OSF under *data/eeg/trf/modulation_model_fit.csv* [52].
(PDF)

S5 Fig Average alpha (8-12 Hz) activity difference between contralateral and ipsilateral central sensors to the cued location (burgundy) and average mu (8–12 Hz; (a) and beta (13–30 Hz; (b) activity difference between contralateral and ipsilateral central sensors to the cued action (green). Horizontal grey lines depict time points that were significantly different between the alpha and mu and the alpha and beta lateralized modulation time courses in a participant-wise cluster-based permutation test. Topographies represent the average mu **(a)** and beta **(b)** activity in contra-vs-ipsi contrasts in informative trials across all sensor pairs during the time-windows which correspond to the mu/beta clusters in Fig 2D. Shaded areas represent the SEM (*N* = 30). The vertical dotted lines represent (from left to right) the offset (0.2 s) of the retro-cue and the time of probe appearance in early trials (1.2 s). The data in this figure can be found in OSF under *data/eeg/trf* [52].
(PDF)

S6 Fig Lack of correlation between the inflection point and minimum shift times of the lateralized alpha and mu/beta modulation time courses. Histograms: the histograms represent the distribution of average mu/beta (green) and alpha (burgundy) minimum shift times **(a)** and minimum inflection points **(b)**, respectively, on all the iterations of the bootstrapping procedure (50 bins per histogram). Centre: scatter plot of the average mu/beta and alpha minimum shift times **(a)** and minimum inflection points **(b)** in each of the 100 bootstrapping iterations across all 30 participants as plotted on the mu/beta (*x*-axis) and alpha (*y*-axis) time space. In **a**, axes are centered around the time of early probe onset. In **a**, vertical and horizontal dotted lines represent the time of early probe appearance (1.2 s after retro-cue onset). The burgundy circles represent the participant-averaged minimum shift time **(a)** and minimum inflection point **(b)** as estimated on the lateralized alpha time course, and the green circles represents the minimum shift time **(a)** and minimum inflection point **(b)** estimated on the lateralized mu/beta activity average. **(a)** The lines on left and bottom the green and burgundy circles, respectively, depict the participant-averaged minimum motor and visual shift times **(a)** and the 95% confidence intervals of the motor and visual inflection points across bootstrapping iterations **(b)**. The lines on right and top of the green and burgundy circles, respectively, depict the participant-averaged maximum motor and visual shift times **(a)** and the 95% confidence intervals of the motor and visual inflection points across bootstrapping iterations **(b)**. Violin plots: Pearson’s *r* values of the correlation between motor and visual minimum shift times **(a)** and minimum inflection point times **(b)** across the 100 bootstrapping iterations for each participant (*N* = 30). Colored dots represent individual participants’ correlation values, the white line inside the grey box represents the mean, and the edges of the grey box represent the first (left) and third (right) quartiles. Schematic representations of the minimum shift time **(a)** and the minimum inflection point **(b)** are on the upper left corner of each scatter plot. Minimum shift time statistics: LMM of minimum visual shift time as a function of minimum motor shift time across bootstrapping iterations, including participant-specific random effects: *β* = −.009, *t* = −.52, *p* = .61. One-sample *t* test between participant-wise correlation values: *t*(29) = −.43, *p* = .67, *d* = .11. Minimum inflection-point statistics: LMM of minimum visual inflection point time as a function of minimum motor inflection point time across bootstrapping iterations, including participant-specific random effects: *β* = −.01, *t* = −.61, *p* = .54. One-sample *t* test between participant-wise correlation values: *t*(29) = −.26, *p* = .8, *d* = .12. The data in this figure can be found in OSF under *data/eeg/trf/boostrap_100.csv* [52].
(PDF)

S1 Methods Supplementary methods. (DOCX)

S1 Table The mean (M) and standard deviation (SD) of reaction time (RT) for different trial types in the behavioral and eeg sessions (*N* = 30). The data in this table can be found in OSF under *figures/behav/ ST1_RT_summary.csv* [52].
(CSV)

S2 Table The mean (M) and standard deviation (SD) of report error for different trial types in the behavioral and eeg sessions (*N* = 30). The data in this table can be found in OSF under *figures/behav/ST2_error_summary.csv* [52].
(CSV)

S3 Table ANOVA results for RT in the behavioral and eeg sessions (*N* = 30). Ges reflects *η*^2^. The data in this table can be found in OSF under *figures/behav/ST3_RT_anova.csv* [52].
(CSV)

S4 Table ANOVA results for report error in the behavioral and eeg sessions (*N* = 30). Ges reflects *η*^2^. The data in this table can be found in OSF under *figures/behav/ ST4_error_anova.csv* [52].
(CSV)

S5 Table The coefficients of the fixed and random effects of the LMM which modelled lateralized alpha and mu/beta modulation as a function of frequency-band and linear, quadratic, and cubic time (see S1 Methods). *N* = 30. The data in this table can be found in OSF under *figures/behav/SupTable5_frequency_modulation_lmer_df.csv* [52].
(CSV)

S1 Data RT values per participant and condition underlying the summary data displayed in Fig 1B. (CSV)

S2 Data Report error values per participant and condition underlying the summary data displayed in Fig 1C. (CSV)

S3 Data Shift time values per participant underlying the summary data displayed in Fig 3C and 3D. (CSV)

S4 Data Pearson’s *r* values per participant underlying the summary data displayed in Fig 4B. (CSV)

## Abbreviations

EEG: electroencephalography
FIR: finite impulse response
ICA: independent component analysis
ITIs: inter-trial intervals
LMM: linear mixed-effects model
