# Supplementary material for: Sensory and motor contents are prioritized dynamically in working memory
Source: PLoS Biol. 2025 Jul 14;23(7):e3003273. doi: 10.1371/journal.pbio.3003273 (PMC12258573; doi:10.1371/journal.pbio.3003273)
Supplement: S1 Fig — (a) Contrast between EEG time–frequency activity contralateral versus ipsilateral to the cued bar location (none) in occipital sensors (PO7, PO8) divided by summed contralateral and ipsilateral activity and expressed as a percentage in noninformative trials. (b) Contrast between EEG time–frequency activity contralateral versus ipsilateral to the cued prospective action (none) in central sensors (C3, C4) divided by summed contralateral and ipsilateral activity and expressed as a percentage in noninformative trials. The first part of the time–frequency spectra (−0.2–1.2 s) corresponds to the average of short and long trials, and the second part (1.2–3.2 s) corresponds to long trials only. The vertical dotted lines represent (from left to right) the onset (0 s) and offset (0.2 s) of the noninformative cue and the time of probe appearance in early trials (1.2 s). No significant clusters were found (N = 30). For comparison purposes, the time–frequency spectra are plotted on the same scale as Fig 2A and 2B. The data in this figure can be found in OSF under data/eeg/trf [52]. (PDF) [file pbio.3003273.s001.pdf]

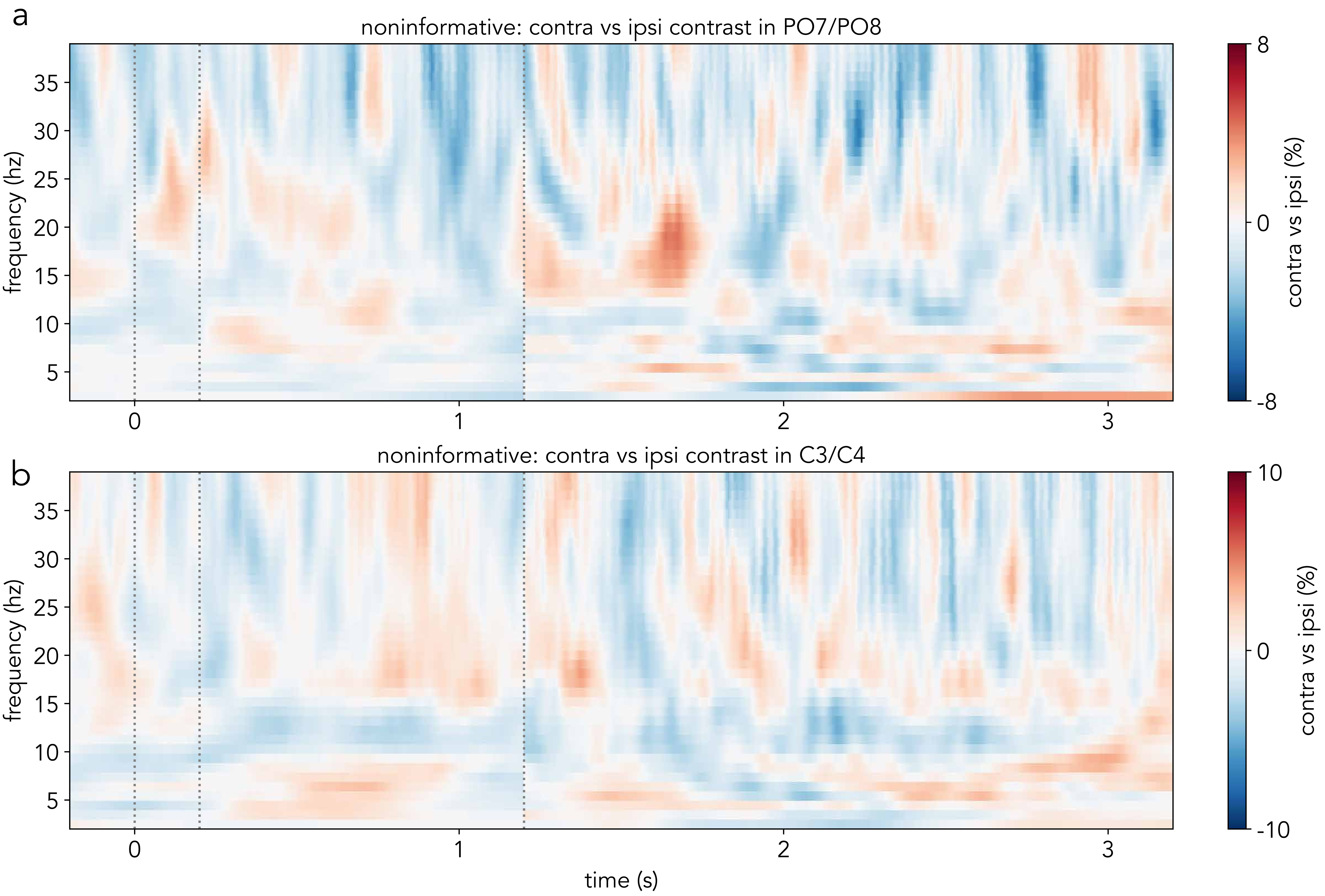
a
noninformative: contra vs ipsi contrast in PO7/PO8
frequency (hz)
35
30
25
20
15
10
5
0
1
2
3
contra vs ipsi (%)
8
0
-8
b
noninformative: contra vs ipsi contrast in C3/C4
frequency (hz)
35
30
25
20
15
10
5
0
1
2
3
time (s)
contra vs ipsi (%)
10
0
-10
