# Supplementary figures and images for: Sensory and motor contents are prioritized dynamically in working memory
Source: PLoS Biol. 2025 Jul 14;23(7):e3003273. doi: 10.1371/journal.pbio.3003273 (PMC12258573; doi:10.1371/journal.pbio.3003273)

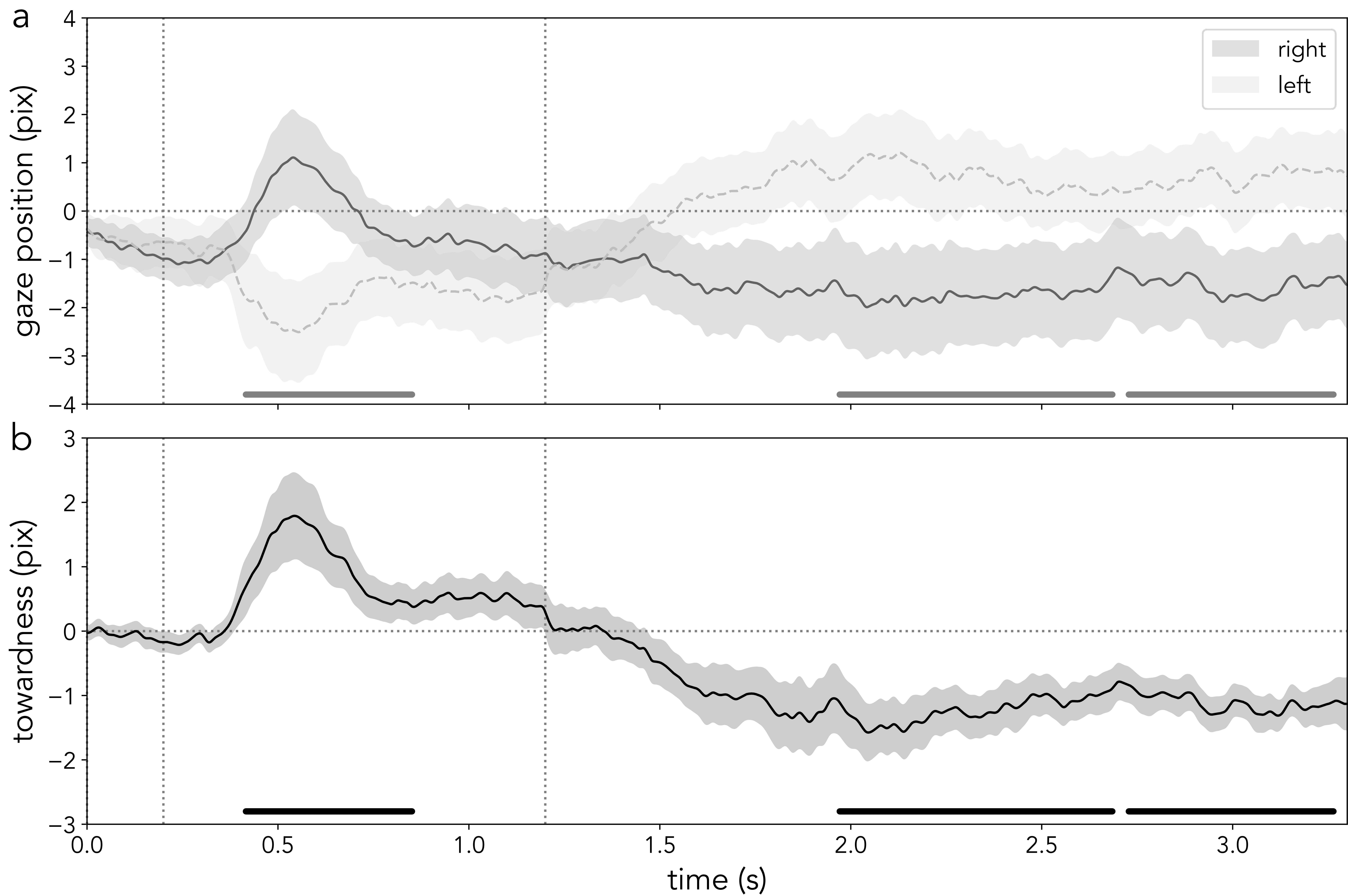

Supplement: S2 Fig — (a) Participant-averaged horizontal gaze position (in pixels) when an item on the left (dotted line) vs on the right (solid line) was cued locked to cue onset in informative trials (first cluster: *p = .01; second cluster: ***p < .001). (b) Participant-averaged towardness (metric which collapses across left and right cued items) locked to cue onset in informative trials (black; first cluster: **p = .009; second cluster: *p = .02). Shaded areas represent the SEM, and vertical dotted lines represent (from left to right) cue offset and time of probe appearance in early trials. Cluster-permutation significant time points are indicated with horizontal lines at the bottom of the plots (N = 30). The first part of the time courses (0–1.2 s) is the average across both short and long trials, and the second part (1.2–3.2) averages across long trials only. The data in this figure can be found in OSF under data/eye [52]. (PDF) [file pbio.3003273.s002.pdf]

**a**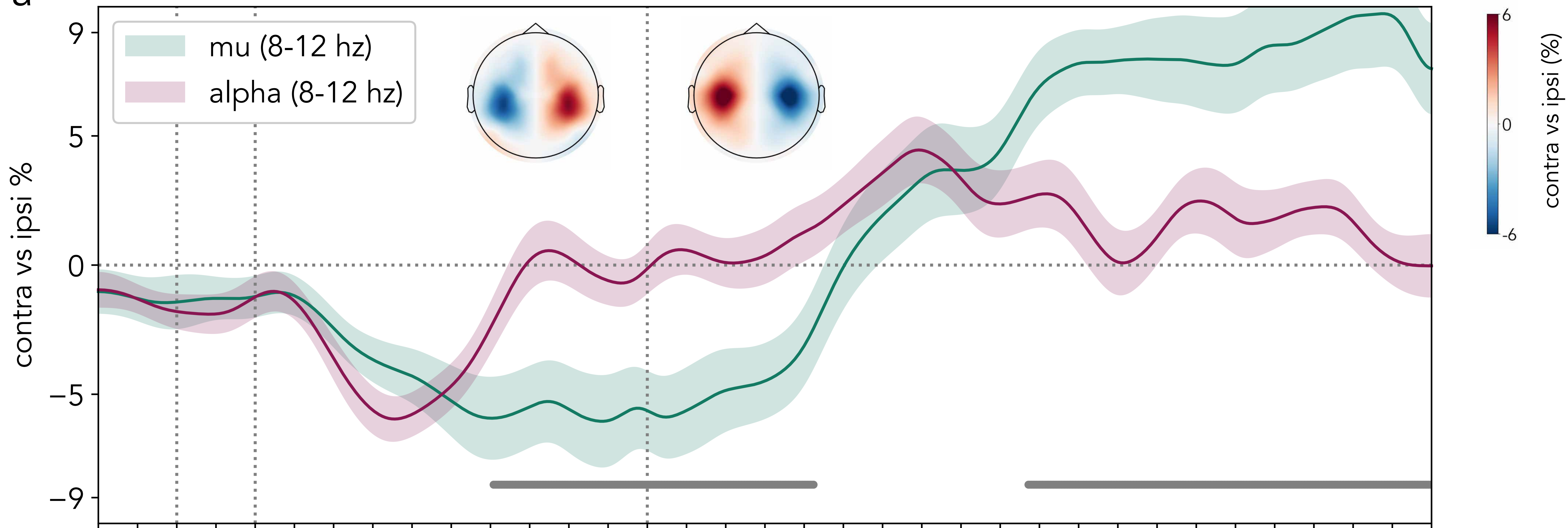**b**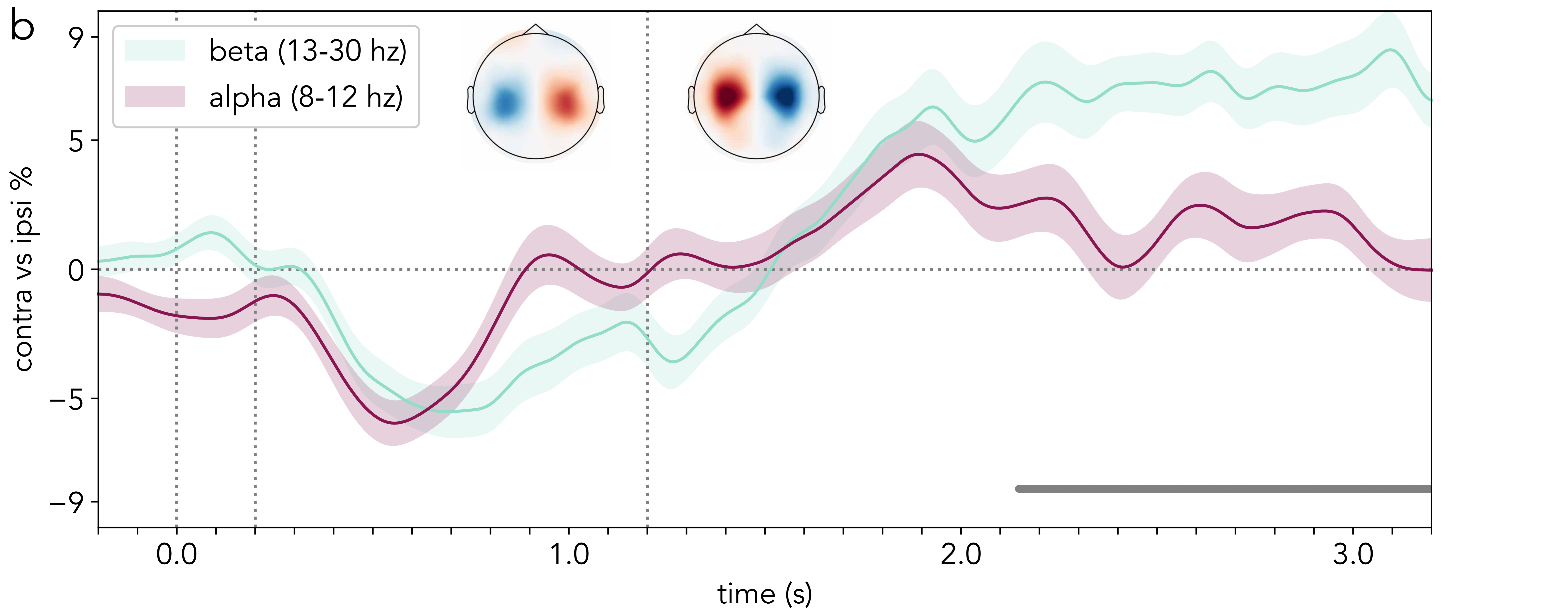

Supplement: S5 Fig — Horizontal grey lines depict time points that were significantly different between the alpha and mu and the alpha and beta lateralized modulation time courses in a participant-wise cluster-based permutation test. Topographies represent the average mu (a) and beta (b) activity in contra-vs-ipsi contrasts in informative trials across all sensor pairs during the time-windows which correspond to the mu/beta clusters in Fig 2D. Shaded areas represent the SEM (N = 30). The vertical dotted lines represent (from left to right) the offset (0.2 s) of the retro-cue and the time of probe appearance in early trials (1.2 s). The data in this figure can be found in OSF under data/eeg/trf [52]. (PDF) [file pbio.3003273.s005.pdf]

a

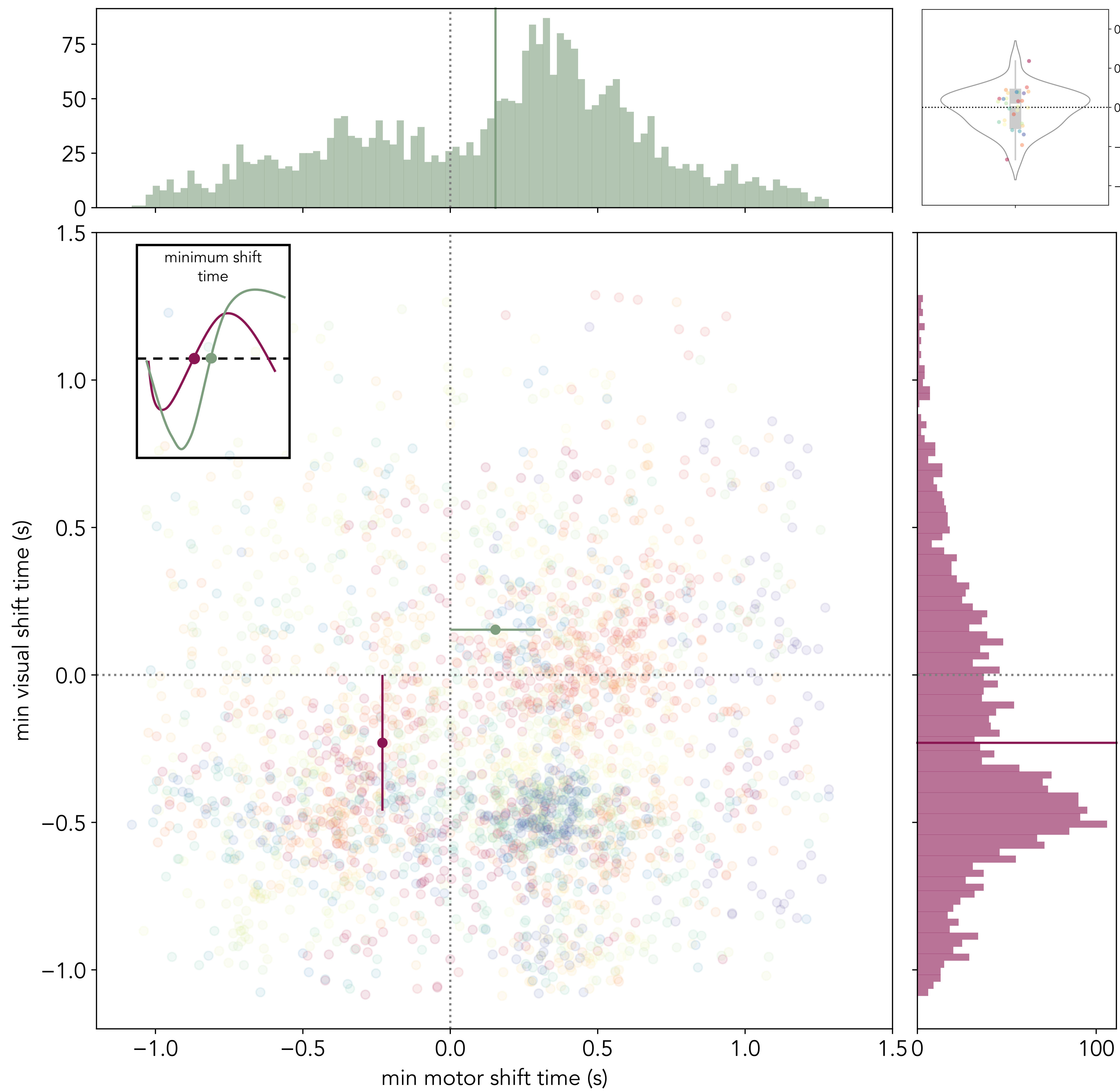

b

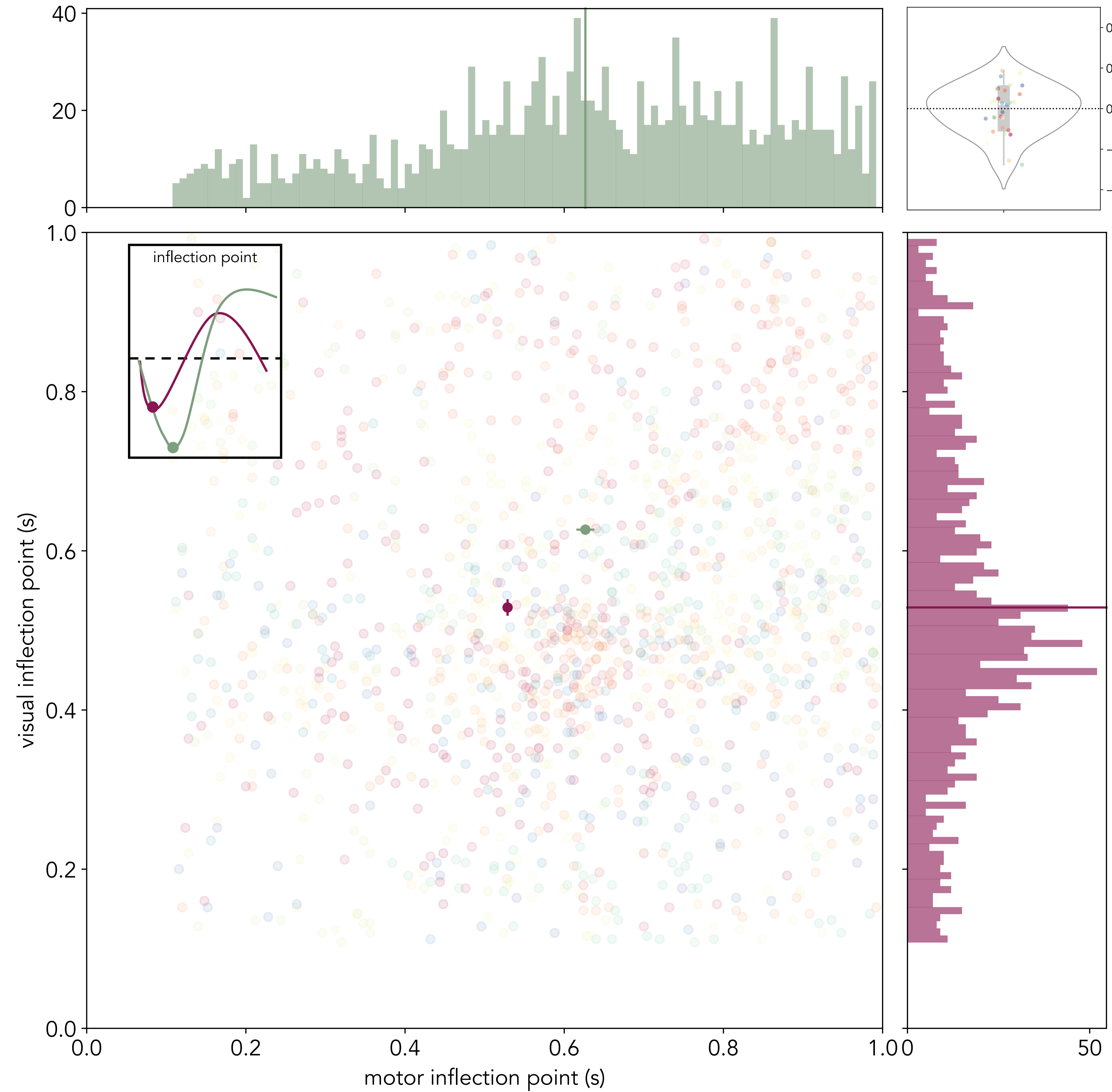

Supplement: S6 Fig — Histograms: the histograms represent the distribution of average mu/beta (green) and alpha (burgundy) minimum shift times (a) and minimum inflection points (b), respectively, on all the iterations of the bootstrapping procedure (50 bins per histogram). Centre: scatter plot of the average mu/beta and alpha minimum shift times (a) and minimum inflection points (b) in each of the 100 bootstrapping iterations across all 30 participants as plotted on the mu/beta (x-axis) and alpha (y-axis) time space. In a, axes are centered around the time of early probe onset. In a, vertical and horizontal dotted lines represent the time of early probe appearance (1.2 s after retro-cue onset). The burgundy circles represent the participant-averaged minimum shift time (a) and minimum inflection point (b) as estimated on the lateralized alpha time course, and the green circles represents the minimum shift time (a) and minimum inflection point (b) estimated on the lateralized mu/beta activity average. (a) The lines on left and bottom the green and burgundy circles, respectively, depict the participant-averaged minimum motor and visual shift times (a) and the 95% confidence intervals of the motor and visual inflection points across bootstrapping iterations (b). The lines on right and top of the green and burgundy circles, respectively, depict the participant-averaged maximum motor and visual shift times (a) and the 95% confidence intervals of the motor and visual inflection points across bootstrapping iterations (b). Violin plots: Pearson’s r values of the correlation between motor and visual minimum shift times (a) and minimum inflection point times (b) across the 100 bootstrapping iterations for each participant (N = 30). Colored dots represent individual participants’ correlation values, the white line inside the grey box represents the mean, and the edges of the grey box represent the first (left) and third (right) quartiles. Schematic representations of the minimum shift time (a) and [file pbio.3003273.s006.pdf]
