# Supplementary material for: Sensory and motor contents are prioritized dynamically in working memory
Source: PLoS Biol. 2025 Jul 14;23(7):e3003273. doi: 10.1371/journal.pbio.3003273 (PMC12258573; doi:10.1371/journal.pbio.3003273)
Supplement: S3 Fig — (a) Contrast between EEG time–frequency activity contralateral versus ipsilateral to the cued bar location in two clusters of lateralized occipital sensors (L: O1, PO7, PO3; R: O2, PO8, PO4) divided by summed contralateral and ipsilateral activity (expressed as a percentage) in informative versus noninformative trials. Black outline indicates significant clusters. (b) Contrast between EEG time–frequency activity contralateral versus ipsilateral to the cued prospective action in two clusters of lateralized central sensors (L: C1, C3, CP1, CP3; R: C2, C4, CP2, CP4) divided by summed contralateral and ipsilateral activity (expressed as a percentage) in informative versus noninformative trials. Black outline indicates statistically significant clusters. (c) Average alpha (8–12 Hz) activity difference between contralateral and ipsilateral sensors to the cued location across participants (burgundy) in informative trials. Average mu/beta (8–30 Hz) activity between contralateral and ipsilateral sensors to the cued action across participants (green) in informative trials. Shaded areas represent the SEM and cluster-based permutation-corrected significant time points are indicated with horizontal lines (burgundy: alpha versus null; green: mu/beta versus null; grey: alpha versus mu/beta; N = 30). The first part of the time–frequency spectra in panels a and b and of the time course in c (−0.2–1.2 s) corresponds to the average of short and long trials, and the second part (1.2–3.2 s) corresponds to long trials only. The vertical dotted lines represent (from left to right) the onset (0 s) and offset (0.2 s) of the retro-cue and the time of probe appearance in early trials (1.2 s). For comparison purposes, the time–frequency spectra are plotted on the same scale as Fig 2. The data in this figure can be found in OSF under data/eeg/trf [52]. (PDF) [file pbio.3003273.s003.pdf]

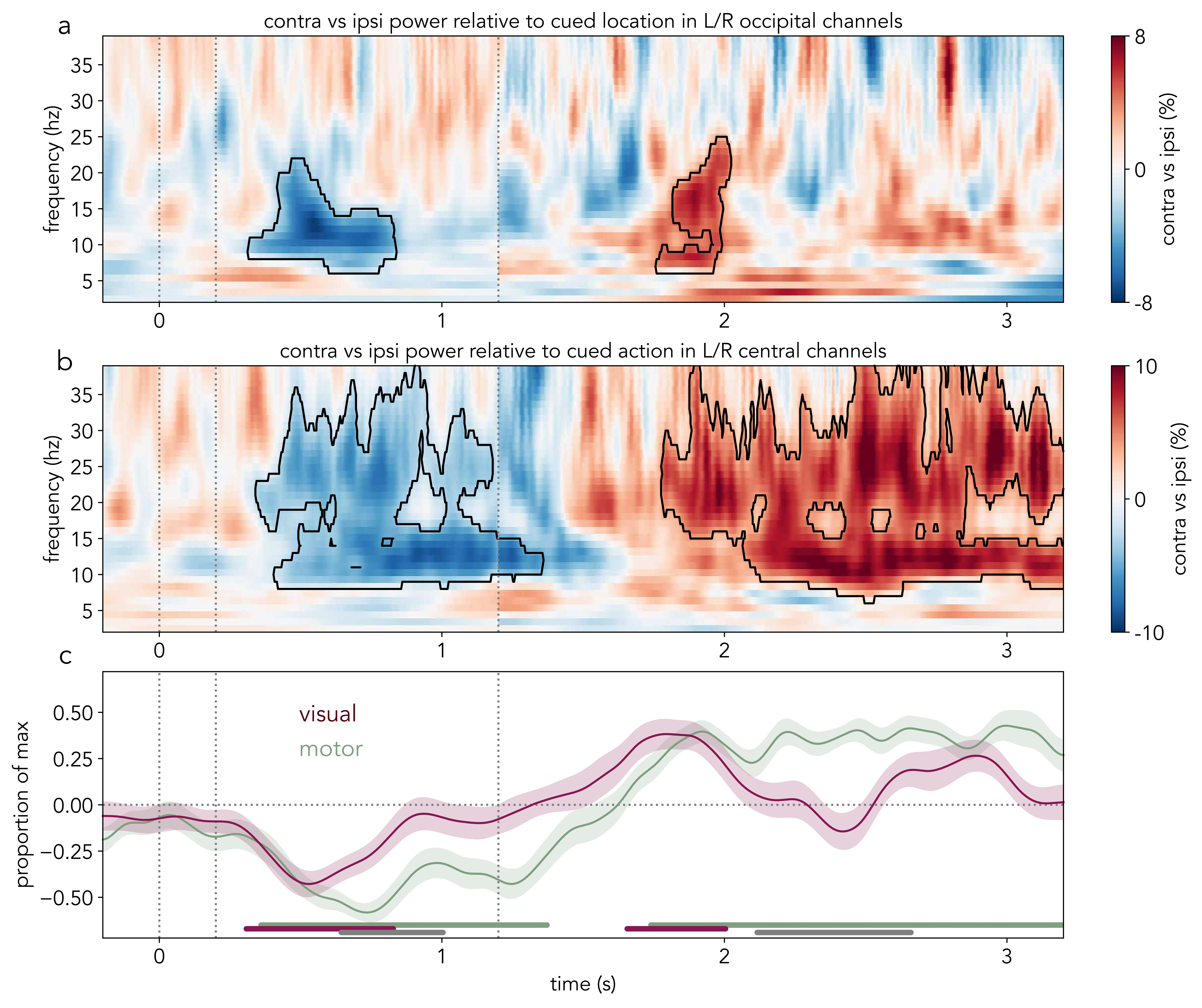
a
contra vs ipsi power relative to cued location in L/R occipital channels
frequency (hz)
contra vs ipsi (%)
8
0
-8
b
contra vs ipsi power relative to cued action in L/R central channels
frequency (hz)
contra vs ipsi (%)
10
0
-10
c
visual
motor
proportion of max
time (s)
0
1
2
3
