# Supplementary material for: Sensory and motor contents are prioritized dynamically in working memory
Source: PLoS Biol. 2025 Jul 14;23(7):e3003273. doi: 10.1371/journal.pbio.3003273 (PMC12258573; doi:10.1371/journal.pbio.3003273)
Supplement: S1 Methods — (DOCX) [file pbio.3003273.s007.docx]

**Supplementary Information**

S1 Methods

**EEG: linear mixed effect model of lateralized alpha and mu/beta modulation**

As a complementary analysis of the temporal coupling between the lateralized alpha and mu/beta modulation time courses, we fit a linear mixed-effects model (LMM; using the *lmer* function in the *lme4* R library) to the participant-averaged lateralized alpha and mu/beta modulation time-course as a function of time and frequency band according to the following formula:

*modulation ~ time** *frequency band* *+ time^2^** *frequency band* *+ time^3^** *frequency band* *+ (1 | participant)*

Here, modulation referred to the participant-averaged contra-vs-ipsi alpha and mu/beta activity modulation in units ranging from -1 (minimum) to 1 (maximum). The frequency-band variable had two levels referring to posterior alpha- (visual) or central mu/beta- (motor) activity respectively. Time represented the time from cue onset until probe onset in long trials centred around the hypothetical onset of the early probe (1.2 s from cue onset). We let the modulation vary as a function of linear, quadratic, and cubic time to capture the non-linear nature of the alpha and mu/beta modulation time courses. Moreover, we let linear, quadratic, and cubic time interact with frequency-band (alpha or mu/beta) to capture linear and non-linear differences in the temporal unfolding of visual and motor prioritization. Finally, we included participant-related random effects in the model. We used the alpha frequency band as the reference contrast. All the coefficients in **S5 Table** ought to be interpreted accordingly. The model was estimated using a maximum likelihood criterion, and the outputs of the model were reported as unstandardised regression coefficients with t-statistics and 99% confidence intervals (**S4 Fig and S5 Table**). We used two-tailed tests and a 5% criterion for significance.

**Eye-tracking data analyses**

In session 2 (EEG), bilateral eye position was continuously monitored with an eye-tracking device at a sampling rate of 1000 Hz (Eyelink 1000, SR-Research Ltd., Ottawa, Ontario, Canada). Participants performed an eye-tracking calibration task before blocks 1, 6, and 11 of the experiment. Additional calibration tasks were performed if ocular drift was noticed between these moments. Calibration did not work for three participants and eye-tracking data could not be collected. One participant was excluded who did not meet the behavioral inclusion criteria. Additionally, three other participants were excluded because the eye-tracking signal was lost for more than 50% of the trials. In total, eye-tracking data from 23 participants were analysed.

The eye-tracking signal was pre-processed following the steps detailed in related studies (58,84,85). First, the acquired edf files were converted into the asc format using EDFConverter (SR-Research Ltd.). All subsequent analyses were performed using Rstudio (75), the *eyelinker* R library, and custom-made scripts.

Eye blinks were identified and the signal +/-100 ms around each blink was discarded following the guidelines from the Eyelink manual (Eyelink 1000, SR-Research Ltd., Ottawa, Ontario, Canada). Subsequently, data from the left and right eye were averaged yielding one time course for eye movements along the horizontal axis (x-position) and another along the vertical axis (y-position). Only the horizontal gaze position was analysed further, as the bars were positioned on the screen along the horizontal axis.

Gaze position data were downsampled to 250 Hz. Next, data were epoched from 500 ms before to 3400 ms after cue onset in long trials and between -500 and 1400 ms in short trials. Trials with eye movements exceeding half the distance to the bars (96 pixels) were removed from further analyses (85). Additionally, trials with RTs below 100 ms or with incorrect responses were discarded from further analyses. An average of 13.05% of trials (SD: 11.15%) were excluded. The time course of the horizontal gaze position in each trial was smoothed using a Gaussian kernel with a standard deviation of 40 ms. Subsequently, the epochs were cropped between 200 ms before and 1200 ms (short) or 3200 ms (long) after the cue to remove any smoothing-related edge artefacts. Finally, the average baseline activity (-200 to 0 ms), was subtracted from each epoch.

Leftward and rightward gaze-position time courses were compared between trials where the left or the right item was cued (**S2A** **Fig**). “Towardness” was calculated as the average between the gaze position in trials where the right item was prioritized and the sign-flipped gaze position in left-item trials. Thus, towardness indicated the extent of biases in gaze position towards the prioritized item location. Cluster-based permutation tested for statistically significant differences between the different time courses.

**EEG: additional visual and motor shift time estimations**

In addition to calculating the average time of zero crossing or lateralization reversal (see *Materials and methods*), we additionally investigated the time of the minimum inflection point and the time of the minimum zero crossing of the lateralized alpha and mu/beta time-courses (see **S6 Fig** for schematics). Following the same bootstrapping procedure detailed above, we tested for a systematic relation between the visual minimum inflection point and motor minimum inflection point and between the minimum visual shift time and the minimum motor shift time across bootstrapping iterations using two LMMs which included participant-related random effects. Moreover, we compared the correlation between visual and motor minimum shift times and minimum inflection point times across bootstrapping iterations per participant with a null hypothesis of no correlation.
